# Selective disruption of trigeminal sensory neurogenesis and differentiation in a mouse model of 22q11.2 deletion syndrome

**DOI:** 10.1242/dmm.047357

**Published:** 2021-05-04

**Authors:** Beverly A. Karpinski, Thomas M. Maynard, Corey A. Bryan, Gelila Yitsege, Anelia Horvath, Norman H. Lee, Sally A. Moody, Anthony-Samuel LaMantia

**Affiliations:** 1 Department of Anatomy and Cell Biology, The George Washington School of Medicine and Health Sciences, Washington DC, 20037, USA; 2 The Fralin Biomedical Research Institute, Virginia Tech Carilion School of Medicine, Roanoke, VA 24014, USA; 3 Department of Pharmacology and Physiology, The George Washington School of Medicine and Health Sciences, Washington DC, 20037, USA; 4 Department of Biological Sciences, Virginia Tech, Blacksburg, VA 24060, USA

**Keywords:** 22q11 deletion syndrome, Cranial places, Neural crest, Precursor proliferation, Sensory neurons, Trigeminal ganglion

## Abstract

22q11.2 Deletion Syndrome (22q11DS) is a neurodevelopmental disorder associated with cranial nerve anomalies and disordered oropharyngeal function, including pediatric dysphagia. Using the *LgDel* 22q11DS mouse model, we investigated whether sensory neuron differentiation in the trigeminal ganglion (CNgV), which is essential for normal orofacial function, is disrupted. We did not detect changes in cranial placode cell translocation or neural crest migration at early stages of *LgDel* CNgV development. However, as the ganglion coalesces, proportions of placode-derived *LgDel* CNgV cells increase relative to neural crest cells. In addition, local aggregation of placode-derived cells increases and aggregation of neural crest-derived cells decreases in *LgDel* CNgV. This change in cell-cell relationships was accompanied by altered proliferation of placode-derived cells at embryonic day (E)9.5, and premature neurogenesis from neural crest-derived precursors, reflected by an increased frequency of asymmetric neurogenic divisions for neural crest-derived precursors by E10.5. These early differences in *LgDel* CNgV genesis prefigure changes in sensory neuron differentiation and gene expression by postnatal day 8, when early signs of cranial nerve dysfunction associated with pediatric dysphagia are observed in *LgDel* mice. Apparently, 22q11 deletion destabilizes CNgV sensory neuron genesis and differentiation by increasing variability in cell-cell interaction, proliferation and sensory neuron differentiation. This early developmental divergence and its consequences may contribute to oropharyngeal dysfunction, including suckling, feeding and swallowing disruptions at birth, and additional orofacial sensory/motor deficits throughout life.

## Abstract (precis)

**Summary:** Altered proportions, distribution, timing and modes of division for trigeminal ganglion progenitors prefigure divergent trigeminal sensory neuron differentiation and oropharyngeal dysfunction in the LgDel mouse model of 22q11.2 deletion syndrome.

## INTRODUCTION

Appropriate genesis and differentiation of cranial sensory neurons is essential for homeostatic behaviors that must be in place at birth in all mammals. The sensory information these neurons transduce and relay, particularly those in the trigeminal ganglion (CNgV), guide key newborn behaviors, including breathing, as well as suckling, feeding and swallowing (S/F/S; Maynard et al., 2020b). Sensation of facial stimuli, as well as mouth, jaw and pharyngeal/laryngeal musculoskeletal activity by CNgV sensory neurons facilitates food intake, optimal nutrition, growth and survival. Early disruption of CNgV differentiation could contribute to suboptimal oromotorotor-motor behaviors in genetic syndromes such as DiGeorge/22q11.2 deletion syndrome (22q11DS; McDonald-McGinn et al., 2015; Meechan et al., 2015a), including pediatric dysphagia, a frequent 22q11DS complication (Eicher et al., 2000; Maynard et al., 2020b). Accordingly, we analyzed CNgV development from midgestation through early postnatal life in *LgDel* mice, a genomically accurate 22q11DS model, in which pups have several deficits that parallel disrupted S/F/S (Karpinski et al., 2016, 2014; Welby et al., 2020; Yitsege et al., 2020).

We investigated whether molecular specification, proliferation and initial differentiation of mechanosensory versus nociceptive sensory progenitors is disrupted as they are generated, respectively, from their placode or neural crest precursors (Ayer-Le Lievre and Le Douarin, 1982; D'Amico-Martel and Noden, 1983; Hamburger, 1961). Neural crest-derived CNgV cells in *LgDel* embryos may be particularly vulnerable to altered differentiation. They arise from anterior hindbrain regions (rhombomeres 2 and 3) that are anomalously patterned to a more posterior identity (Karpinski et al., 2014; Maynard et al., 2020a). Thus, neural crest migration and coalescence into CNgV may be altered. In addition, local signaling between neural crest-derived sensory ganglion cells (Wakamatsu, 2011; Wakamatsu et al., 2000) or placode- versus neural crest-derived cells (Shiau and Bronner-Fraser, 2009; Shiau et al., 2008; Steventon et al., 2014; Wu and Taneyhill, 2019) may be disrupted due to divergent specification of distinct CNgV precursors. These changes, presumably due to diminished 22q11 gene dosage in the hindbrain or in CNgV cells themselves (Karpinski et al., 2014; Maynard et al., 2003, 2020a; Meechan et al., 2006), could compromise subsequent axon growth, target innervation or functional differentiation leading to aberrant transduction, relay or transmission of orofacial sensory information and subsequent oropharyngeal dysfunction, including pediatric dysphagia.

*LgDel* mouse pups fail to gain weight, aspirate milk and develop naso-sinus, middle ear and respiratory infections (Karpinski et al., 2014; LaMantia et al., 2016; Maynard et al., 2020b; Yitsege et al., 2020), similar to perinatal dysphagia, which may reflect aberrant CN V sensory innervation or function. Adult *LgDel* mice have craniofacial anomalies and oro-motor behavioral deficits that indicate continued difficulties with feeding and swallowing circuits that include CN V (Welby et al., 2020). We have now identified disrupted precursor proliferation, distribution and modes of division for distinct lineage-related cell classes during early CNgV development in the *LgDel*. These early changes prefigure differences in the identity of CNgV sensory neurons in neonatal animals that could contribute to aberrant orofacial sensory-motor coordination, particularly for circuits that contribute to dysphagic symptoms during early life in *LgDel* mice.

## RESULTS

### Initial assembly of *LgDel* CNgV

**Fig. 1.**
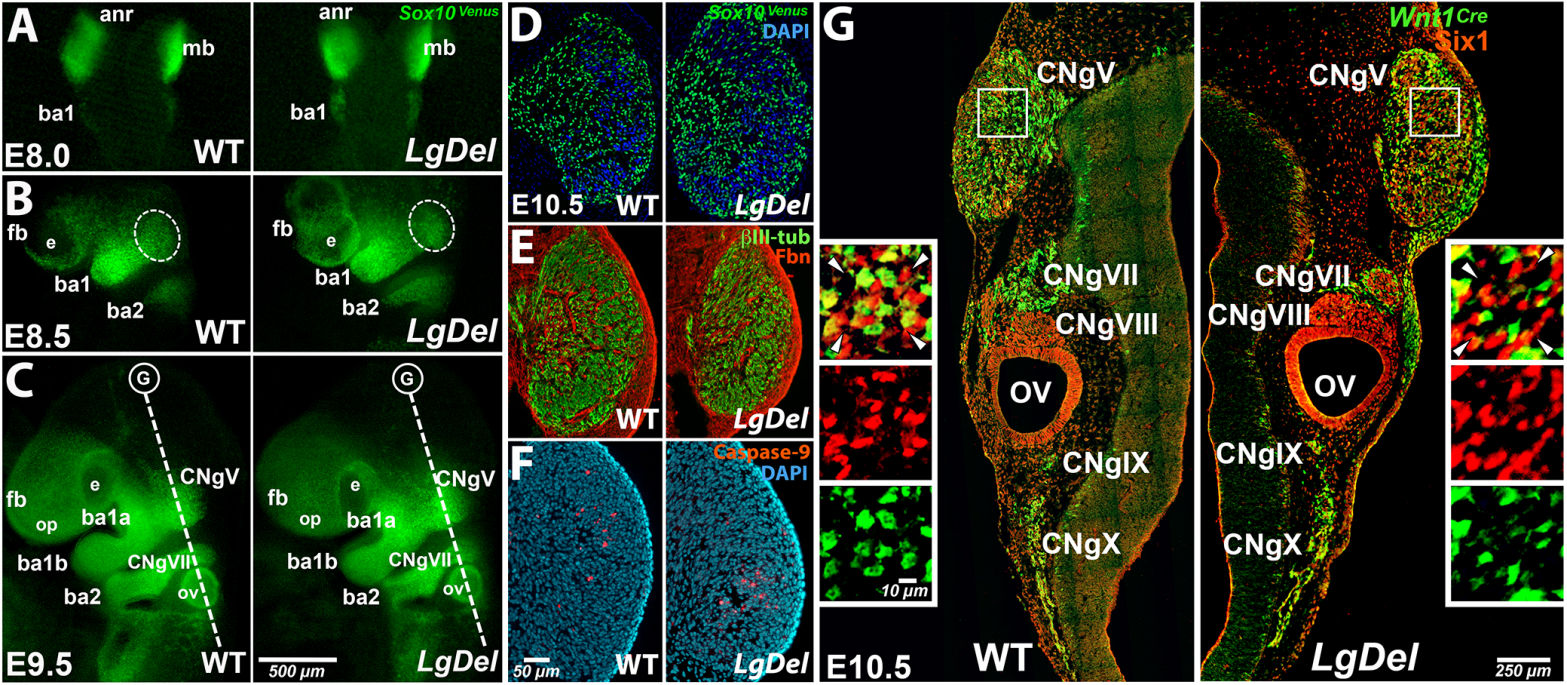
**The CNgV in wild-type and *LgDel* embryos.** (A) *Sox10^V^*^enus+^ midbrain (mb) neural crest cells were seen in the open anterior neural tube, posterior to the anterior neural ridge (anr) at E8.0 in both genotypes and in the branchial arch 1 (ba1). (B) By E8.5, as the neural tube closes, the forebrain (fb), eye (e) and cranial mesenchyme were distinct in both genotypes. *Sox10^V^*^enus+^ cells were seen in the branchial arch mesenchyme (ba1 and ba2), and the region in which CNgV will coalesce (dashed ovals). (C) By E9.5 in both genotypes, cranial neural crest cells were seen in all typical locations: surrounding the fb, adjacent to the olfactory placode (op) and otic vesicle (ov), and segregating into a defined CNgV. By this age, ba1 has become subdivided into the maxillary anlagen (ba1a) and mandibular anlagen (ba1b), and the adjacent CNgV is becoming distinct from these ba1 subdivisons. The dashed line indicates the plane of the section shown in G and subsequent analyses. (D) At E10.5, in both genotypes, *Sox10^V^*^enus+^ cells have coalesced into a distinct CNgV. (E) Fibronectin (Fbn) was excluded from CNgV at E10.5, except for around penetrating blood vessels, and βIII-tubulin^+^ neurons were seen throughout. (F) At E10.5, cleaved caspase 3^+^ cells were sparse in central CNgV in both genotypes. (G) At E10.5, all cranial ganglia were recognizable in *LgDel*, in comparable positions to wild-type (WT) counterparts. Mosaic composition of placode (Six1^+^) and neural crest (*Wnt1^Cre^*^+^) cells was apparent in both genotypes for CNgV, CNgVII, CNgIX and CNgX; the spiral ganglion (CNgVIII) in wild type and *LgDel* had primarily Six^+^, presumably placode-derived cells. In *LgDel* E10.5 CNgV, Six1^+^ cells appeared to be more frequent and seemed slightly more aggregated than in wild type (box, insets, arrowheads).

We first assessed changes in the distribution of migratory hindbrain neural crest cells to CNgV or altered coalescence of placode- and neural crest-derived cells within CNgV. We used a *Sox10^Venus^* reporter (Corpening et al., 2011; Shibata et al., 2010) to visualize migrating neural crest in embryonic day (E)8.0, E8.5 and E9.5 *LgDel* and wild-type embryos (Fig. 1A-C). In E8.0 embryos [open anterior neural tube, corresponding to Theiler stage (TS)12], we saw no apparent difference in the density or distribution of *Sox10^Venus^*^+^ cells between the hindbrain and facial primordia, where CNgV will coalesce (Fig. 1A). Similarly, there were no grossly detectable differences at E9.0 (forebrain/branchial arch 1A/B visible, equivalent to TS13; Fig. 1B) when neural crest cells begin to accumulate where CNgV will form. Finally, *Sox10^Venus^*^+^ cell density and distribution remained indistinguishable in E9.5 wild-type and *LgDel* embryos when CNgV is recognizable as a focal cellular aggregate (equivalent to TS14; Fig. 1C). Thus, the schedule and pattern of neural crest migration is not appreciably altered by 22q11 deletion during early cranial differentiation.

We next investigated whether *LgDel* CNgV at E10.5, after the ganglion coalesces, has normal boundaries separating it from the surrounding mesenchyme, or differs noticeably in shape, size or cytological organization. We evaluated sections through CNgV from E10.5 *Sox10^Venus^* wild-type and *LgDel* embryos, and found no substantial differences in CNgV boundaries or coherence (Fig. 1D). To further evaluate cytological differentiation of CNgV from the surrounding mesenchyme in *LgDel*, we assessed the distribution of fibronectin, which in wild type was largely excluded from CNgV, being expressed only around blood vessels within wild-type CNgV (Fig. 1E). A similar pattern of fibronectin expression was seen in *LgDel* CNgV at E10.5 (Fig. 1E). Using a complete series of fibronectin-labeled sections to trace CNgV boundaries, we quantified wild-type and *LgDel* ganglion volumes (see Materials and Methods). We found no difference in E10.5 *LgDel* versus wild-type CNgV volume (wild type=0.035 mm^3^, *n*=4; *LgDel*=0.033mm^3^, *n*=5; *P*=0.55). However, we noticed that the local distribution of neural crest-derived cells (Fig. 1D), and presumed newly generated neurons (Fig. 1E), within CNgV seemed different in *LgDel*. Thus, we calculated cell density by counting all CNgV cells (labeled by DAPI; Fig. 1F) in multiple sections of wild-type and *LgDel* CNgV. Despite apparently similar volumes, there was a significant (34%) decline in CNgV cell density at E10.5 based upon this parallel sample of *LgDel* versus wild-type embryos (wild type: 111±11 cells/100um^2^, *n*=8 ganglia/6 embryos/4 litters; *LgDel*: 73±2 cells/100um^2^, *n*=6 ganglia/4 embryos/2 litters; *P*≤0.009). We cannot confidently calculate CNgV cell numbers based on independent volume and cell density samples; nevertheless, comparison of the values suggest differences. To evaluate whether such differences reflect substantially altered cell death in *LgDel*, perhaps parallel to that we found due to pharmacologically altered Fgf signaling in wild type (Karpinski et al., 2016), we assessed cleaved caspase 3^+^ cell frequency and saw no apparent divergence between genotypes (Fig. 1F).

**Fig. 2.**
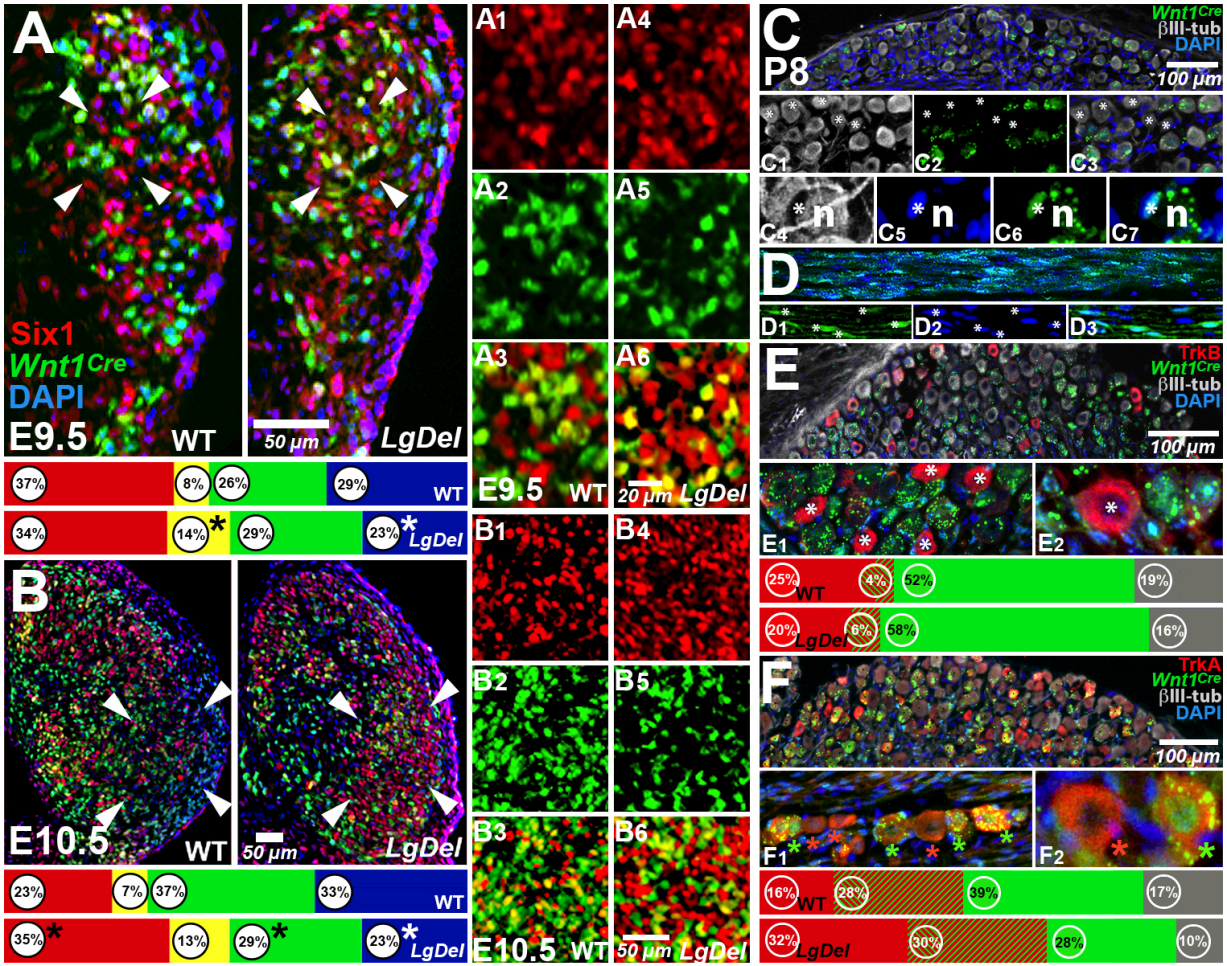
**Divergent cell classes in developing and early postnatal *LgDel* CNgV.** (A) At E9.5, Six1^+^ (placode), *Wnt1^Cre^*^+^ and DAPI^+^ (neural crest) were seen in both genotypes. The frequency of Six^+^/*Wnt1^Cre^*^+^ cells was higher in *LgDel* CNgV (*P=0.0009, *t*-test) and the frequency of DAPI^+^ cells was lower (*P=0.02, *t*-test). The arrowheads on the left indicate the location of the region enlarged in A1-A3, and those on the right indicate the location of the region enlarged in A4-A6. At E9.5, wild-type (WT) *Wnt1^Cre^*^+^ cells were intermixed with Six1^+^ cells (A1-A3), whereas Six1^+^ cells appeared to be differentially aggregated in *LgDel* (A4-A6). (B) At E10.5, Six1^+^ versus *Wnt1^Cre^*^+^ and DAPI^+^ proportions continued to change. Asterisks indicate significant differences in *LgDel* (*P=0.017; Six1^+^, 0.01; *Wnt1^Cre^*^+^, 0.035, DAPI^+^; *t*-test). The arrowheads on the left indicate the location of the region enlarged in B1-B3, and those on the right indicate the location of the region enlarged in B4-B6. Six1^+^ and *Wnt1^Cre^*^+^ cells in wild type appeared to be more intermixed (B1-B3), and there were local aggregates of Six1^+^ cells in *LgDel*. (C) Preservation of lineage distinctions in wild-type and *LgDel* P8 CNgV. Subsets of CNgV sensory neurons (gray, βIII-tubulin^+^; C1) were *Wnt1^Cre^*^+^ (C2,C3), as were satellite glia (C3-C7). In C1-C3, the asterisks indicate locations of βIII-tubulin^+^ sensory neurons that are not *Wnt1^Cre^*^+^. In C4-C7, the asterisks indicate single *Wnt1^Cre^*^+^ satellite cells, and 'n' indicates the sensory neuron, labeled by βIII-tubulin in C3, of which the *Wnt1^Cre^*^+^ cell is a satellite. (D) Axon fascicles within P8 CNgV had subsets of *Wnt1^Cre^*^+^ (D1, asterisks) and *Wnt1^Cre^*^−^ (D2-D3) Schwann cells. (E) TrkB^+^ presumably mechanosensory neurons (asterisks, E1,E2) were present at low frequencies in P8 wild-type and *LgDel* CNgV. Histograms show proportions of TrkB^+^ (red), TrkB^+^/*Wnt1^Cre^*^+^ (red/green), *Wnt1^Cre^*^+^ (green) and βIII-tubulin^+^-only (gray) sensory neurons (*n*=189 cells, wild type; 268 cells, *LgDel*; 1 wild-type and 1 *LgDel* P8 pup). (F) TrkA^+^ presumably nociceptive sensory neurons were *Wnt1*^Cre+^ (green asterisks, F1,F2) and *Wnt1*^Cre−^ (red asterisks, F1,F2). Histograms show proportions of TrkA^+^, TrkA^+^/*Wnt1^Cre^*^+^, *Wnt1^Cre^*^+^ and *Wnt1^Cre^*^−^ sensory neurons (βIII-tubulin) in wild type and *LgDel* (*n*=333 cells, wild type; 279 cells, *LgDel*; 1 wild-type and 1 *LgDel* P8 pup).

We recently reported an increase in the proportions of placode-derived cells (Six1^+^) and a corresponding decline of neural crest-derived cells identified by lineage tracing using *Wnt1^Cre^*^/+^/*Rosa26-GNZ^Tg/+^* (denoted as *Wnt1^Cre^*^+^, Fig. 2) in *LgDel* E10.5 CNgV (Maynard et al., 2020a). To assess whether these two distinct populations are noticeably altered in additional cranial ganglia, we compared their distribution in *LgDel* and wild-type E10.5 CNgV, geniculate ganglia (CNgVII), spiral ganglia (CNgVIII), glossopharyngeal (CNgIX) and vagal ganglia (CNgX). These ganglia are present in both genotypes in similar positions relative to the otic vesicle (Fig. 1G). CNgV, VII, IX and X have both Six1^+^ and *Wnt1^Cre^*^+^ cells, whereas CNgVIII and the otic vesicle epithelium are populated primarily by Six1^+^ cells. In *LgDel* versus wild type, Six1^+^ cells seemed more frequent relative to *Wnt1^Cre^*^+^ cells in CNgV VII, IX and X (Fig. 1G), and their distribution appeared to be distinct (Fig. 1G, insets). Six1^+^ cells appeared slightly more aggregated, with fewer *Wnt1^Cre^*^+^ cells interspersed. Thus, although neural crest migration and placodal cell ingression were maintained in *LgDel*, once these cells coalesced into a recognizable CNgV, their spacing and distribution appeared to be altered.

### Divergence of progenitor classes in *LgDel* CNgV

Distinct proportions of placode- versus neural crest-derived cells in *LgDel* CNgV at E10.5 (Maynard et al., 2020a) could be a result of increased numbers of placode-derived cells or a delayed or diminished arrival of neural crest cells in the ganglion as they coalesce between E9.0 and E9.5. Thus, we determined relative frequencies of Six1^+^, *Wnt1^Cre^*^+^, Six1^+^/*Wnt1^Cre^*^+^, and Six1^−^/*Wnt1^Cre^*^−^/DAPI^+^-only cells (‘DAPI^+^’) in wild type versus *LgDel* E9.5 CNgV (Fig. 2A). The ‘DAPI^+^-only’ cells represent a subset of Foxd3^+^/Six1^−^ presumably neural crest cells not included in the *Wnt1^Cre^* transcriptional lineage (Karpinski et al., 2016; Maynard et al., 2020a); we refer to them as ‘DAPI^+^’ for convenience. At E9.5, there was no significant difference in the proportions of Six1^+^ cells (*P*=0.17, *t*-test) or *Wnt1^Cre^*^+^ cells (*P*=0.28, *t*-test; *LgDel*, *n*=9 ganglia, 7 embryos, 4 litters; wild type, *n*=7 ganglia, 4 embryos, 3 litters); however, the proportion of *LgDel* Six1^+^/*Wnt1^Cre^*^+^ cells increased significantly (*P*=0.0009, *t*-test) and the proportion of DAPI^+^ cells decreased significantly (*P*=0.02, *t*-test). Finally, at E9.5, Six1^+^ cells appeared to be differentially segregated from neural crest neighbors in *LgDel* (arrowheads; Fig. 2A,A1-A6). Apparently, there is an initial proportional divergence and difference in the distribution of distinct E9.5 CNgV cell classes associated with placode- and neural crest-derived populations (Karpinski et al., 2016).

As reported previously (Maynard et al., 2020a), *LgDel* versus wild-type placode versus neural crest proportions diverge by E10.5 (Fig. 2B; *n*=11 ganglia, 8 embryos, 6 litters; wild type, 9 ganglia, 5 embryos, 4 litters; Six1^+^: *LgDel*>WT, *P*=0.017; *Wnt1^Cre^*^+^: *LgDel*<WT, *P*=0.01; DAPI^+^: *LgDel*<WT, 0.035; *t*-test). In wild type, CNgV cells expressing neural crest-associated markers predominated by E10.5. However, in *LgDel*, the proportions of placode- and neural crest-associated cell classes were nearly identical to those at E9.5. There were significantly larger proportions of *LgDel* CNgV Six1^+^ cells, significantly smaller proportions of *Wnt1^Cre^*^+^ and DAPI^+^ cells, and no change in Six1^+^/*Wnt1^Cre^*^+^ cells with uncertain placode versus neural crest origins. The apparent differential segregation of Six1^+^ versus *Wnt1^Cre^*^+^ and DAPI^+^ cells (arrowheads; Fig. 2B,B1-B6) remains in *LgDel* at E10.5, perhaps due to proportional changes in each cell class. Apparently, between E9.5 and E10.5, *LgDel* CNgV differentiation favors placode versus neural crest cells, which may enhance anomalous segregation of cell classes.

### CNgV sensory neuron identities and transcriptional lineage in wild-type and *LgDel* CNgV

To confirm that *Wnt1^Cre^* lineage tracing identifies differentiated cranial sensory neurons with neural crest origins, we analyzed *Wnt1^Cre^*^+^ cells in postnatal day (P)8 wild-type and *LgDel* CNgV. These observations are based upon the assessment of two P8 pups of each compound genotype. Limited numbers of these P8 mice precludes statistical comparison between genotypes. Accordingly, we present these data to evaluate coincidence of markers, but not to define statistical divergence in wild type and *LgDel*. There is a substantial subset of *Wnt1^Cre^*^+^/βIII-tubulin^+^ sensory neurons in wild-type as well as *LgDel* P8 CNgV (Fig. 2C,C1-C3), as well as subsets of *Wnt1^Cre^*^+^ satellite cells (Fig. 2C4-C7) and Schwann cells (Fig. 2D). Thus, *Wnt1^Cre^*^+^ neural crest progenitors give rise to differentiated CNgV sensory neurons and peripheral glia in both wild type and *LgDel*.

To further assess the association of *Wnt1^Cre^^+^* cells with presumed mechanoreceptive versus nociceptive cells, we evaluated co-labeling of P8 CNgV *Wnt1^Cre^*^+^/βIII-tubulin^+^ neurons with the neurotrophin receptors TrkB or TrkA (also known as Ntrk2 and Ntrk1, respectively) in sections from one P8 *Wnt1^Cre^*:WT and one P8 *Wnt1^Cre^*:*LgDel* mouse pup (*n*=189 cells wild type; 268 cells *LgDel* for TrkB/βIII-tubulin; *n*=333 cells wild type, 279 cells *LgDel* for TrkA/βIII-tubulin). The association of TrkB with mechanoreceptors and TrkA with nociceptors is not absolute. There is evidence for TrkB signaling in nociceptors (Perez-Pinera et al., 2008; Shu and Mendell, 1999; Wang et al., 2009) and TrkA^−^ nociceptors have been reported previously (Golden et al., 2010; Alshawaf et al., 2018). Our data indicate similar proportions of TrkB^+^/, TrkA^+^/ and *Wnt1^Cre^*^+^/βIII-tubulin^+^ sensory neurons in the two genotypes; however, we cannot compare these proportions statistically due to the limited sample. TrkB^+^ CNgV sensory neurons were less frequent than TrkA^+^ sensory neurons (29% TrkB^+^, wild type; 26%, TrkB^+^, *LgDel*; 44% TrkA^+^, wild type, 62% TrkA^+^, *LgDel*; Fig. 2E, see also Fig. 7). TrkB^+^/βIII-tubulin^+^ cell proportions approximate E10.5 Six1^+^ cell frequency, and the small subset of TrkB^+^/*Wnt1^Cre^*^+^/βIII-tubulin^+^ cells in both genotypes may reflect the small population of Six1^+^/*Wnt1^Cre^*^+^ E10.5 progenitors (Fig. 2E). The remaining TrkB^−^ CNgV sensory neurons in wild type (71%) and *LgDel* (74%) were mostly *Wnt1^Cre^*^+^ with a minority of βIII-tubulin^+^-only cells, perhaps related to DAPI^+^ progenitors (Fig. 2E). Approximately two-thirds (64%) of wild-type TrkA^+^ sensory neurons versus 48% of *LgDel* TrkA+ sensory neurons were *Wnt1^Cre^*^+^. The remaining βIII-tubulin^+^ CNgV sensory neurons were mostly *Wnt1^Cre^*^+^. Together, these data suggest that the proportions of presumed mechanoreceptive and nociceptive CNgV sensory neurons in wild-type and *LgDel* P8 pups parallel the distribution of related placode- and neural crest-associated progenitor cell classes in CNgV in E10.5 wild-type and *LgDel* embryos.

### Altered neighbor relationships of CNgV placode versus neural crest precursors

**Fig. 3.**
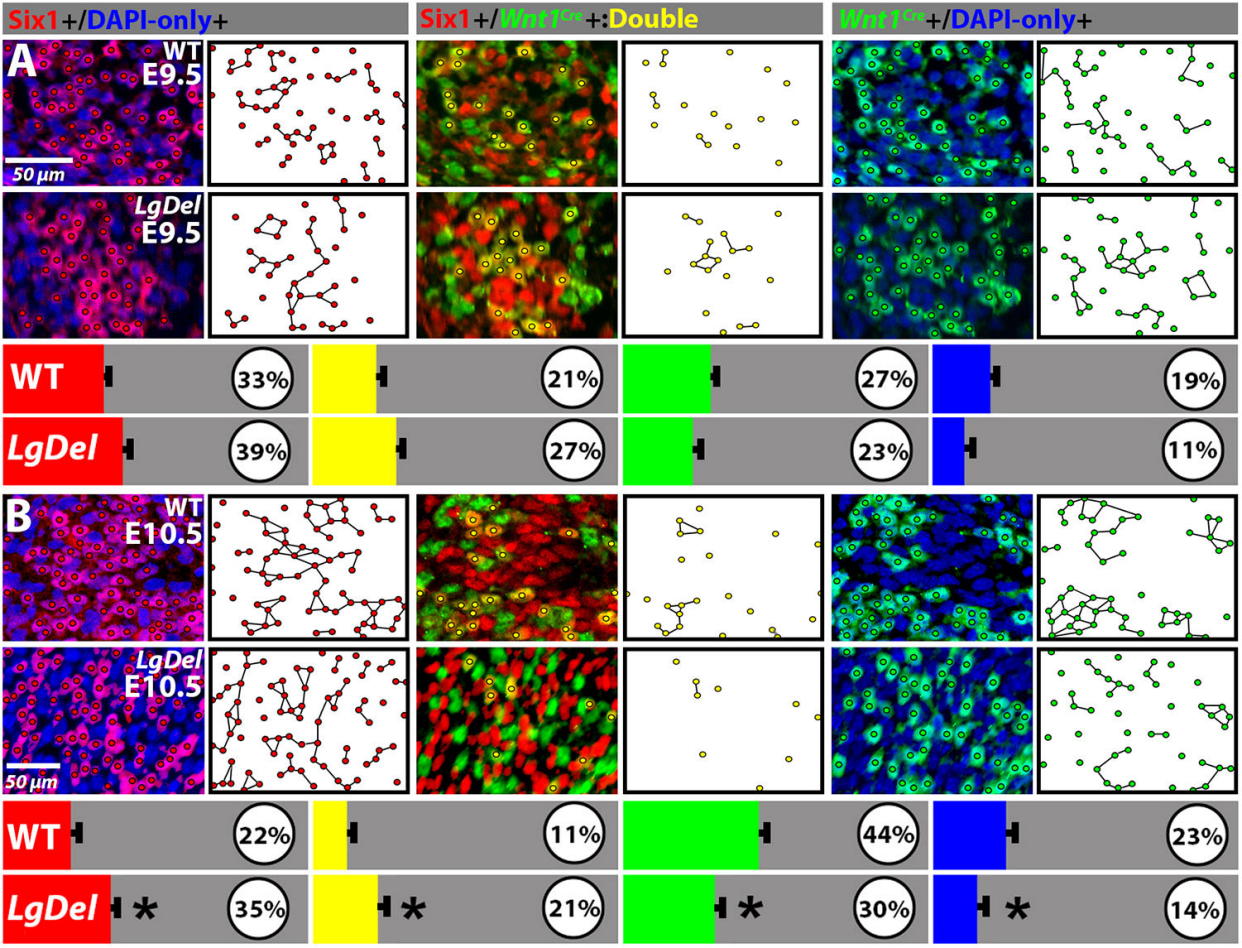
**Neighbor relations and class-selective aggregation of CNgV cells during initial CNgV differentiation.** (A) Top two rows: representative images of E9.5 wild-type (WT, top row) or *LgDel* (second row) Six1^+^ and DAPI^+^, Six1^+^/*Wnt1^Cre^*^+^, or *Wnt1^Cre^*^+^ and DAPI^+^ CNgV cells (left panels), and representative plots (right panels) showing groups of cells shown on the left. Cells joined by line segments have cells of similar classes as immediate neighbors. Third row: percentages of cells of each class with at least one cell of the same class as a neighbor (cells immediately adjacent). At E9.5, these percentages did not differ significantly in wild type versus *LgDel*. (B) E10.5 wild-type (top row) or *LgDel* (second row) Six1^+^ and DAPI^+^, Six1^+^/*Wnt1^Cre^*^+^, or *Wnt1^Cre^*^+^ and DAPI^+^ CNgV cells (left panels), and plots (right panels). Cells joined by line segments have cells of similar classes as immediate neighbors. The histograms (third row) show percentages of cells of each class that had at least one cell of the same class as a neighbor. In the wild-type E10.5 CNgV, percentages had shifted. Neural crest-associated cells (*Wnt1^Cre^*^+^ and DAPI^+^) had a significantly greater percentage of like neighbors than their E9.5 counterparts. In contrast, this shift was not seen in *LgDel* E10.5 CNgV. In *LgDel* CNgV, the proportions of Six1^+^ cells and Six1^+^/*Wnt1^Cre^*^+^ cells with like neighbors increased (*P<0.0001 and *P*<0.0004, respectively; two-way ANOVA), and proportions of *Wnt1^Cre^*^+^ and DAPI^+^ cells declined (*P*<0.0001 and *P*<0.008, respectively; two-way ANOVA).

Apparent changes in the distribution and proportions of distinct cell classes in the coalescing *LgDel* CNgV suggest that 22q11 deletion alters local neighbor relationships between cells of like versus distinct lineages. To assess these relationships, which might influence cell-cell interactions, we developed an algorithm to evaluate the proportions of each population with neighbors of similar molecular identity in multiple serial sections of entire wild-type and *LgDel* CNgVs (see Materials and Methods). In the E9.5 wild-type CNgV (*n*=4 ganglia) ∼one-fifth to one-third of each class (Six1^+^, Six1^+^/*Wnt1^Cre^*^+^, *Wnt1*^Cre+^, and DAPI^+^) had cells of the same type as neighbors (Fig. 3A). These values for each class did not differ significantly in *LgDel* CNgV (*n*=7 ganglia; two-way ANOVA). Apparently, CNgV in both genotypes is a mosaic with limited preferential aggregation of any cell class. By E10.5, in wild-type CNgV (*n*=9 ganglia), proportions of three of the four cell classes changed significantly from E9.5: Six1^+^ and Six1^+^/*Wnt1^Cre^*^+^ cells had fewer like neighbors (*P*<0.0001 and *P*<0.02, respectively; two-way ANOVA); *Wnt1^Cre^*^+^ cells had more like neighbors (*P*<0.007); and DAPI^+^ cells with like neighbors remained unchanged. This change was not observed in E9.5 versus E10.5 *LgDel* CNgV (*n*=11 ganglia). In *LgDel* CNgV, Six1^+^ and Six1^+^/*Wnt1*^Cre+^ cells had significantly larger proportions of like cell neighbors than wild type (*P*<0.0001 and *P*<0.0004, respectively), and the proportion of *Wnt1^Cre^*^+^ and DAPI^+^ cells with like neighbors declined (*P*<0.0001 and *P*<0.007, respectively; Fig. 3B; see also Fig. 2A1-A6). Apparently, wild-type CNgV Six1^+^ cells acquire more diverse neighbors than *LgDel*, perhaps due to differences in proliferation or terminal neurogenesis of placodal versus neural crest precursors (D'Amico-Martel and Noden, 1983).

### Informatic assessment of CNgV differential cell adhesion/interaction gene expression

Changes in *LgDel* CNgV neighbor relations, expression of multiple 22q11 genes in the wild-type ganglion and their 50% decrement in *LgDel* (Karpinski et al., 2016; Maynard et al., 2020a) suggest that 22q11 deletion may dysregulate genes that mediate cell-cell interactions. Thus, we re-analyzed the expression of genes and splice isoforms involved in cell-cell adhesion and signaling using a previously generated RNA-seq dataset of E10.5 wild-type and *LgDel* CNgV transcriptomes from five biological replicates of pooled ganglia from multiple embryos and litters (Maynard et al., 2020a). We defined several sets of cell adhesion or cell-cell interaction genes: Notch and related factors (Siebel and Lendahl, 2017); Connexin (*Gja*) gap junction subunits (Swayne and Bennett, 2016); *Cxcl* ligands and the *Cxcr4* receptor (Kawaguchi et al., 2019); Wnt ligands/receptors (Steinhart and Angers, 2018); and cadherin (Cdh)/catenin (Ctn) mediators (Brault et al., 2001; Shiau and Bronner-Fraser, 2009; Wu and Taneyhill, 2019). Consistent with our expression level analysis for all wild-type versus *LgDel* CNgV transcripts (Maynard et al., 2020a), 22/22 Notch-related, 10/12 Gja, and 3/4 Cxcl/Cxcr genes had higher coefficients of variation (CVs) in *LgDel* versus wild-type CNgV; however, none were differentially expressed (Fig. S1A). Of the *Wnt*/*Cdh*-related genes, 28/38 had *LgDel*>WT CVs, and Wnt/Cdh/Cat transcripts had 4: *Wnt1*, *Wnt3a*, *Fzd3* and *Fzd10* were differentially expressed (*LgDel*<WT; Fig. S1A). However, *Wnt1* and *Wnt3a* were expressed at low levels. In parallel CVs of 12/14 genes associated with CNgV neurogenesis and maturation were greater in *LgDel*. Increased variation suggests 22q11 deletion destabilizes gene expression for cell-cell interactions and neuronal differentiation.

Using Leafcutter software (Li et al., 2018), we found significant enrichment of differential splicing in *LgDel* CNgV, including four genes associated with neuronal morphology and proliferation (Fig. S1B): *Adgrg1* (also known as GPR56) (Salzman et al., 2016) associated with polymicrogyria; *adgrv1* (also known as GPR98 and VLGR1) (McMillan et al., 2002) with Usher Syndrome; L1-related Ig-like *Nfasc* (Ebel et al., 2014) with neural development and demyelinating neuropathies; and *Cd151* (*tetraspanin*) was implicated in trigeminal placode identity and placodal sensory neuron differentiation (McCabe et al., 2004). The 5′UTR variants for *Adgrg1* and *Cd151* were enriched in *LgDel*, as is an *adgrv1* nonsense-mediated decay variant (Fig. S1C). An *Nfasc* in-frame exon retention presumably led to a protein coding variant (Fig. S1C). Thus, divergent variable expression and splicing of adhesion-related genes accompanies changes in cell position and neighbor identities in *LgDel* CNgV.

### Cell proliferation in *LgDel* CNgV

**Fig. 4.**
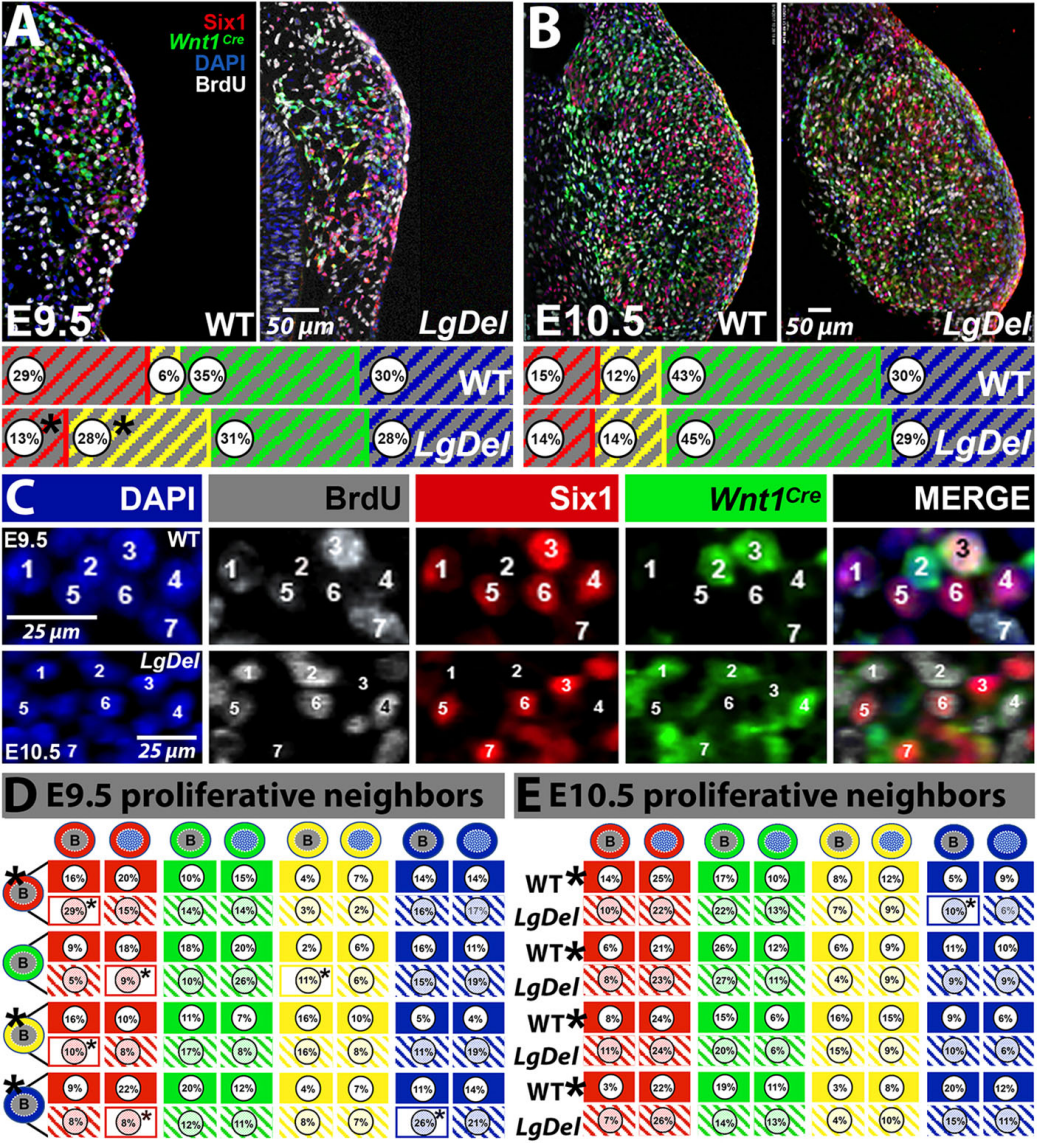
**Proliferative activity of molecular/lineage distinct progenitor classes in E9.5 and E10.5 wild-type and *LgDel* CNgV.** (A) At E9.5, subsets of wild-type (WT) and *LgDel* Six1^+^, *Wnt1^Cre^*^+^ and DAPI^+^ cells were acutely labeled by BrdU (white). The histograms show that in *LgDel* the proportion of Six1^+^/BrdU^+^ cells declined significantly at E9.5, whereas the proportion of Six1^+^/*Wnt1^Cre^*^+^/BrdU cells increased (*n*=3 ganglia, 3 embryos, 3 litters, wild type; 3 ganglia, 3 embryos, 2 litters, *LgDel*; *P≤0.0018, Six1^+^/BrdU, *P≤0.006, Six1^+^/*Wnt1^Cre^*^+^, *t*-test). (B) At E10.5, there were no detectable differences in the proportions of CNgV BrdU^+^, as well as Six1^+^, Six1^+^/*Wnt1^Cre^*^+^, *Wnt1^Cre^* and DAPI^+^ cells. (C) Identities and distribution of individual BrdU^+^ cells across all cell classes in E9.5 (wild type), as well as E10.5 (*LgDel*) CNgV. (D,E) Neighbor analysis of proliferative cells (cell icons with ‘B’ in the nucleus, far left) of each class at E9.5. The row of cell icons (top, D,E) shows the array of potential neighbors that BrdU^+^ cells of each class can have. The percentage of BrdU^+^ cells of each class that have neighbor cells for each possible neighbor pairing is shown in the individual panes: solid panes provide wild-type values and hatched panes provide *LgDel* values. Panes that are outlined rather than solid or hatched are individual BrdU proliferative progenitor/neighbor pairings that are significantly different between wild type and *LgDel* (E9.5). (D) Six1^+^/BrdU^+^:Six1/BrdU^+^, *P*<0.005; Six1^+^/BrdU^+^:Six1^+^/*Wnt1^Cre^*^+^:BrdU^+^, *P*<0.0001; *Wnt1^Cre^*^+^/BrdU^+^:Six1^+^, *P*<0.025; *Wnt1^Cre^*^+^/BrdU^+^:Six1^+^/*Wnt1^Cre^*^+^/BrdU^+^, *P*<0.03; DAPI^+^/BrdU^+^:Six1^+^, *P*<0.0002; DAPI^+^/BrdU^+^:DAPI^+^/BrdU^+^, *P*<0.0002. ANOVA with Tukey post-hoc correction. Asterisks to the upper right of BrdU^+^ cell classes (far left columns, D,E) indicate significant differences of neighbor identity across all possible pairings (*P*<0.0001 for all significantly different categories; two-way ANOVA/Holm–Sidak).

Cellular and gene expression changes in developing *LgDel* versus wild-type CNgV suggest *LgDel* versus CNgV precursors may be differentially proliferative. Thus, we quantified CNgV cells acutely in S-phase, labeled via maternal injection of BrdU 2 h before collecting E9.5 or E10.5 wild-type and *LgDel* embryos (Fig. 4A). CNgV cells of all categories incorporated BrdU at E9.5 and E10.5; however, there was a pronounced bias toward labeling neural crest cells in both genotypes at the two ages (Fig. 4A-C), consistent with early terminal division and differentiation of placode-derived cranial sensory neurons, and enhanced proliferation of neural crest precursors at mid-gestation (D'Amico-Martel, 1982; D'Amico-Martel and Noden, 1983). At E9.5, the proportion of Six1^+^/BrdU^+^ cells decreased by ∼twofold in *LgDel* (*P*=0.006, *t*-test), whereas the proportion of Six1^+^/*Wnt1^Cre^*^+^/BrdU^+^ cells increased nearly fourfold (*P*=0.001; Fig. 4A; wild type: *n*=3 ganglia, 3 embryos, 3 litters; *LgDel*: 3 ganglia, 3 embryos, 2 litters). The latter increase was consistent with the significant increase of *LgDel* CNgV Six1^+^/*Wnt1^Cre^*^+^ cells at E9.5 (see Fig. 2A). However, by E10.5, proportions of BrdU^+^ cells in each class were statistically indistinguishable in *LgDel* and wild type (wild type: *n*=4 ganglia, 3 embryos, 2 litters; *LgDel*: *n*=4 ganglia, 3 embryos, 2 litters; Fig. 4B). The return to equivalent Six1^+^/BrdU^+^ cell frequency in E10.5 *LgDel* CNgV suggests that proliferation of either placodal or neural crest progenitors is disproportionately altered between E9.5 and E10.5. However, it is unclear whether this reflects accelerated early proliferation among *LgDel* placode progenitors, or retarded proliferation among *LgDel* neural crest progenitors.

Differences in the proliferative capacity of *LgDel* versus wild-type Six^+^ and Six1^+^/*Wnt1^Cre^*^+^ cells at E9.5 versus E10.5 may be accompanied by altered neighbor relations between proliferative and non-proliferative cells. We therefore compared the frequencies of BrdU^+^ cells of each class with their BrdU^+^ versus BrdU^−^ neighbors at E9.5 and E10.5 in wild-type and *LgDel* CNgV (Fig. 4D,E). Of the 32 possible individual neighbor comparisons, five pairs involving progenitors that express Six1 and one paring within the DAPI^+^ cell class were significantly different between the two genotypes (wild type: *n*=3 ganglia, 3 embryos, 3 litters; *LgDel*: 3 ganglia, 3 embryos, 2 litters; ANOVA with Tukey post-hoc; Fig. 4D). In contrast, there are no significant frequency differences of any individual neighbor pairs in E10.5 *LgDel* versus wild-type CNgV (Fig. 4E). However, we noticed that the overall distribution of frequency values for neighbor pairings across each of the BrdU^+^ actively proliferating progenitor classes seemed divergent in the two genotypes. Indeed, the distribution of neighbor frequencies for E9.5 Six1^+^/BrdU^+^, *Wnt1^Cre^*^+^/BrdU^+^ and DAPI^+^/BrdU^+^ proliferative progenitors differs significantly in *LgDel* versus wild-type CNgV (*P*<0.0001 for each cell class). By E10.5, neighbor relations for all four proliferative precursor classes differed significantly in *LgDel* versus wild-type CNgV (*P*<0.0001 for each cell class). Apparently, there are divergent proportions, interactions and timings of placode and neural crest proliferative progenitors in *LgDel* versus wild-type CNgV.

### Altered neurogenic yield for *LgDel* neural crest

**Fig. 5.**
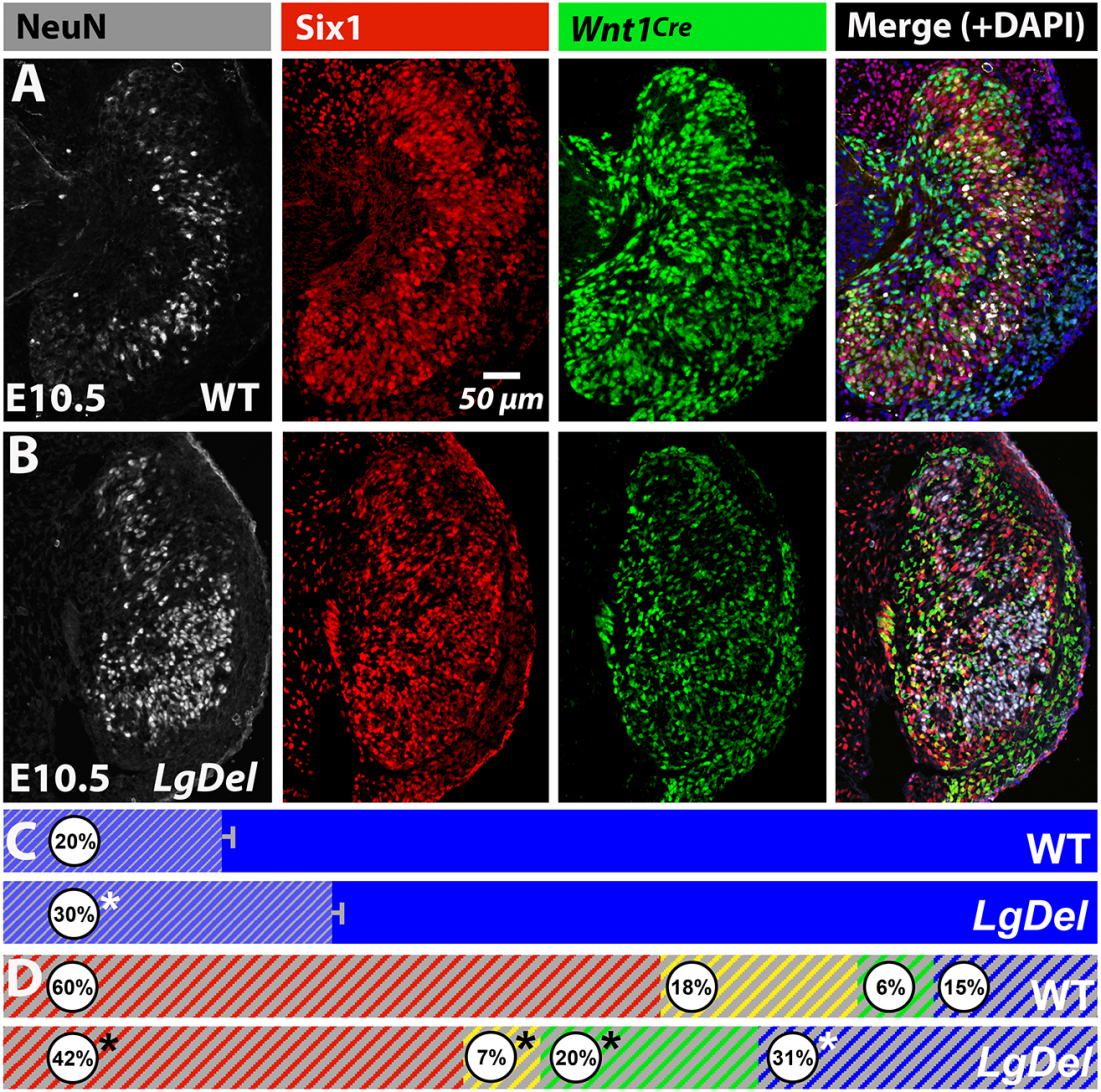
**Increased neurogenic yield in E10.5 *LgDel* CNgV.** (A) Wild-type (WT) E10.5 CNgV labeled for NeuN (RbFox3), which recognizes newly postmitotic neurons. NeuN^+^ neurons accumulated in the periphery of wild-type CNgV, in which Six1^+^ cells were also segregated by this age. (B) *LgDel* E10.5 CNgV NeuN^+^ neurons and their relation to Six1^+^, *Wnt1^Cre^*^+^ and DAPI^+^ cells; NeuN^+^ cells appeared to be more numerous and more widely distributed. (C) NeuN^+^ neuron frequency relative to all cells increased significantly in *LgDel* E10.5 CNgV (*n*=5 ganglia, 4 embryos, 2 litters, wild type; 4 ganglia, 4 embryos, 3 litters, *LgDel*; *P<0.00015, *t*-test). (D) Proportions of NeuN^+^ neurons in wild type and LgDel that are Six1^+^, Six1^+^/*Wnt1^Cre^*^+^, *Wnt1^Cre^*^+^ and DAPI^+^. The increased neurogenic yield in *LgDel* E10.5 CNgV reflected a disproportionate increase in NeuN^+^ cells within the *Wnt1^Cre^*^+^ and DAPI-only subpopulations (*P≤0.015, Six1^+^; 0.0029, Six1^+^/ *Wnt1^Cre^*^+^; 0.0054, *Wnt1^Cre^*^+^; 0.011, DAPI^+^, *t*-tests).

Differences in proliferation, as well as neighbor relationships of lineage-related *LgDel* CNgV cell classes at E9.5 versus E10.5, indicates that the schedule and frequency of neurogenesis may be altered. Accordingly, we assessed the proportions of early differentiating neurons – the neurogenic yield – based on NeuN (also known as RbFox3) expression (Duan et al., 2016) in *LgDel* versus wild-type CNgV at E10.5, when placode- versus neural crest-derived cell proportions diverge. The numbers and distribution of NeuN^+^ cells differed in E10.5 *LgDel* versus wild-type CNgV (Fig. 5A,B). In wild type, NeuN^+^ cells were segregated in the lateral CNgV (Fig. 5A), whereas in *LgDel*, they were distributed more broadly (Fig. 5B). The total frequency of NeuN^+^ cells as a percentage of all cells (total number of DAPI-labeled nuclei independent of any other label) increased significantly in E10.5 *LgDel* versus wild-type CNgV (Fig. 5C; *LgDel*, 30±1%; wild type, 20±1%; *P*≤0.0001; *n*=5 ganglia, 4 embryos, 2 litters; wild type, *n*=4 ganglia, 4 embryos, 3 litters).

It seemed possible that proportions of NeuN^+^ cells that were also Six1^+^, Six1^+^/*Wnt1^Cre^*^+^, *Wnt1^Cre^*^+^ or DAPI^+^ might vary between the two genotypes. Such changes would suggest divergent neurogenic capacities of distinct progenitor classes in *LgDel* versus wild-type CNgV. Accordingly, we analyzed proportions of NeuN^+^ cells in wild-type and *LgDel* CNgV associated with placodal- and neural crest-associated cell classes (Fig. 5D). In *LgDel*, NeuN^+^/Six1^+^ cell frequency declined by 18%, and NeuN^+^/Six1^+^/*Wnt1^Cre^*^+^ frequency declined by 11% (*P*≤0.015, Six1^+^; 0.0029, Six1^+^/*Wnt1^Cre^*^+^). In contrast, NeuN^+^/*Wnt1^Cre^*^+^ cell frequency tripled, and NeuN^+^/DAPI^+^ cell frequency doubled (*P*≤0.0054, *Wnt1^Cre^*^+^; 0.011, DAPI). Apparently, the increased total number of NeuN^+^ cells in *LgDel* CNgV cells reflects increased terminal neurogenesis of neural crest progenitors by E10.5. This change, perhaps combined with anomalously accelerated early terminal neurogenesis in Six1^+^ and Six1^+^/*Wnt1^Cre^*^+^ progenitors at E9.5, may result in the shifted proportions of placode- versus neural crest-associated CNgV cells in *LgDel*.

### Increased asymmetric terminal neurogenic division in *LgDel* CNgV

**Fig. 6.**
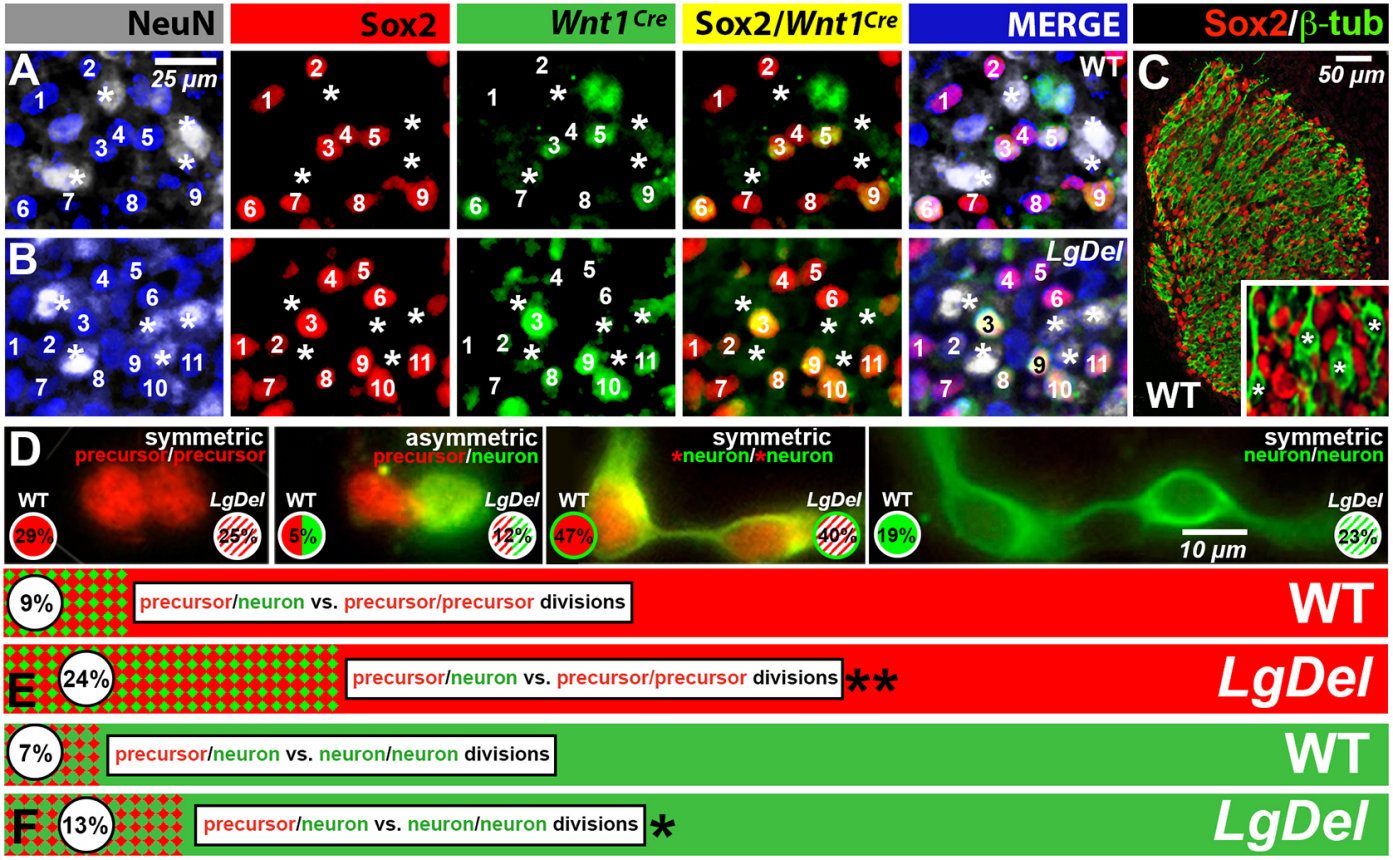
**Increased neurogenic yield reflects altered proportions of asymmetric neurogenic CNgV precursor divisions.** (A) Sox2^+^ cells in wild-type (WT) E10.5 CNgV were not NeuN^+^, but included a subset of *Wnt1^Cre^*^+^ cells. (B) Sox2^+^ cells in *LgDel* E10.5 CNgV were not NeuN^+^, and included a subset of *Wnt1^Cre^*^+^ and DAPI^+^ cells, as in wild type. The asterisks indicate the positions of NeuN^+^ presumed neurons, which are coincident with either Sox2^+^ or *Wnt1^Cre^*^+^ CNgV cells. (C) Sox2 and βIII-tubulin-labeled cells were distinct populations in E10.5 CNgV. Inset: Sox2^+^ nuclei were adjacent to differentiating neurons, the processes of which were labeled by βIII-tubulin (asterisks). (D) Distinct modes of CNgV progenitor division in pair cell assays from dissociated E10.5 wild-type or *LgDel* CNgV, plated at low density, after 24 h *in vitro*. The proportion of precursor divisions of each mode/genotype are given in the lower left (wild type) and lower right (*LgDel*). (E) There was a significant increase in the proportion of asymmetric versus symmetric divisions among *LgDel* CNgV precursors (wild type: *n*=373 pairs, 22 ganglia/11 embryos/5 litters; *LgDel*: 226 pairs, 14 ganglia/7 embryos/4 litters, **P=0.0045, Fisher's exact test). (F) There was a corresponding significant increase in the proportion of asymmetric neurogenic divisions among *LgDel* CNgV precursors (*P=0.039, Fisher's exact test).

Increased neurogenesis among neural crest cells and changes in neighbor relations among distinct progenitor classes suggests that modes of cell division (Gotz and Huttner, 2005), rather than rates or levels of proliferation, may be altered for *LgDel* CNgV neural crest precursors. Thus, we quantified self-renewing versus neurogenic divisions of E10.5 *LgDel* and wild-type CNgV precursors using a pair-cell assay (Fig. 6). In these assays, we used Sox2 to identify neural progenitors (Pevny and Nicolis, 2010) and βIII-tubulin to identify early neurons (Fanarraga et al., 1999; Moody et al., 1989). Sox2 was expressed primarily in *Wnt1^Cre^*^+^ or DAPI^+^ cells in both wild-type and *LgDel* E10.5 CNgV *in vivo* (Fig. 6A,B; wild type, 73%; *LgDel*, 71%), only rarely in NeuN^+^ cells (1% in both genotypes) and was excluded from βIII-tubulin^+^ cells *in vivo* (Fig. 6C). We used Sox2/βIII-tubulin labeling *in vitro* to enhance the resolution of nuclear (Sox2) versus cytoplasmic/process labeling (βIII-tubulin) in cells of each pair. The pair cell assay identified three modes of CNgV precursor divisions (Fig. 6D): progenitor-progenitor (Sox2^+^:Sox2^+^); progenitor-neurogenic (Sox2^+^:βIII-tubulin^+^); and symmetric neurogenic (Sox2^+^/βIII-tubulin^+^:Sox2^+^/βIII-tubulin^+^ and βIII-tubulin^+^:βIII-tubulin^+^). The proportional frequency of symmetric progenitor-progenitor versus asymmetric progenitor-neurogenic divisions differed significantly between wild-type and *LgDel* CNgV cells. There were nearly threefold more asymmetric neurogenic divisions in *LgDel* pairs (*LgDel*, 24% asymmetric; wild type, 9% asymmetric; *P*<0.0045, Fisher exact test; Fig. 6E). In parallel, the proportion of *LgDel* asymmetric versus symmetric neurogenic divisions increased significantly (*LgDel*, 13% asymmetric; wild type, 7% asymmetric; *P*<0.04, Fisher exact test; Fig. 6F). These changes suggest a higher neurogenic yield via asymmetric division in *LgDel* CNgV, consistent with increased NeuN^+^ postmitotic neuron frequency generated primarily by neural crest progenitors.

### Postnatal sequelae of divergent neurogenesis in *LgDel* CNgV

**Fig. 7.**
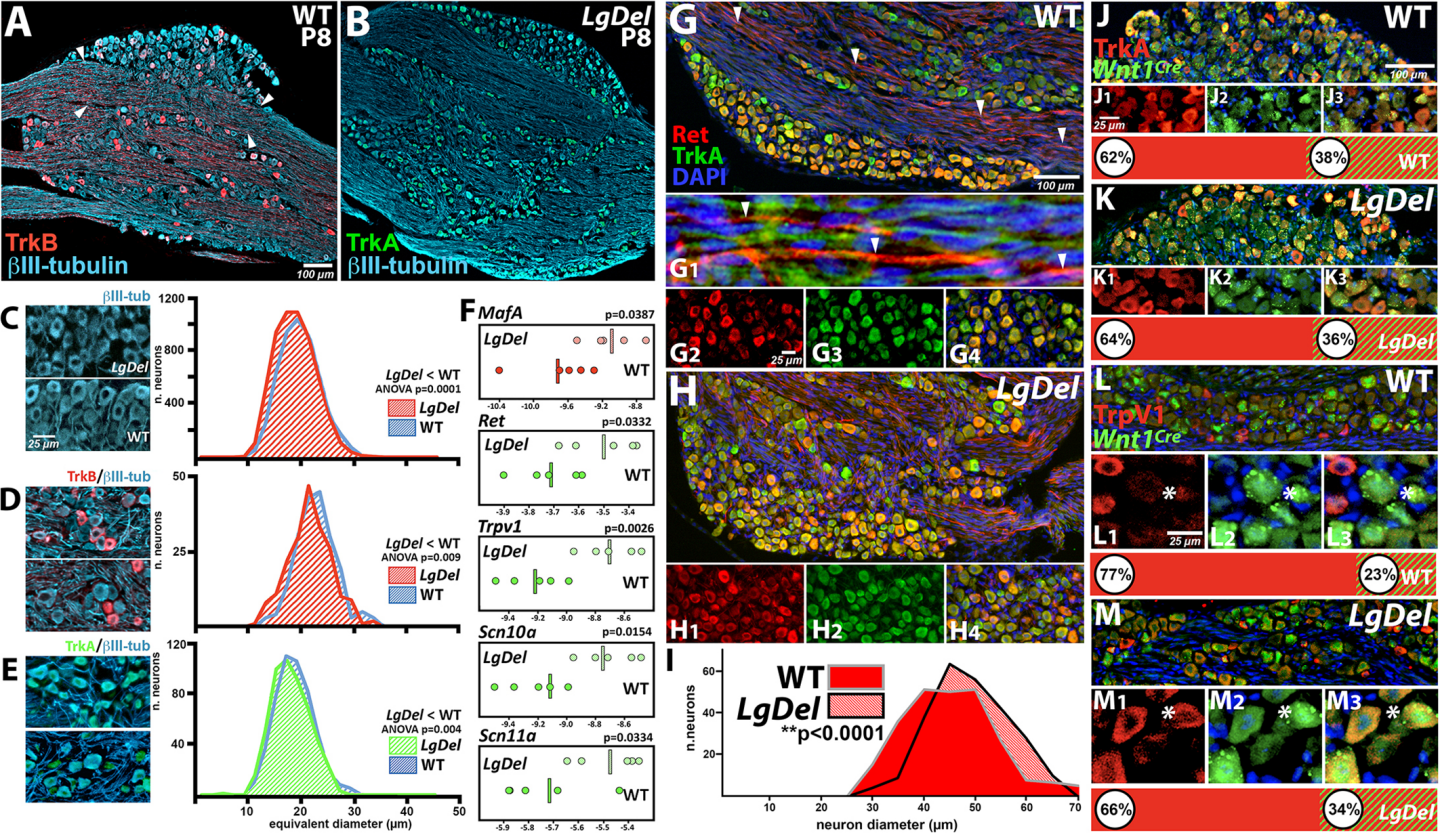
**Altered sensory neuron differentiation in *LgDel* P8 CNgV.** In panels A and B, single examples of wild-type (WT) and *LgDel* sections used for quantification presented in panels C, D and E are shown. (A) P8 CNgV had βIII-tubulin^+^ sensory neurons (blue) in nodules separated by bundles of CN V axons that included TrkB^+^ glia and axons (red, arrowheads). TrkB^+^ neurons appeared to be a minority (see also Fig. 2). (B) TrkA^+^ cells (green) accounted for a majority of βIII-tubulin^+^ sensory neurons at P8. (C) P8 *LgDel* CNgV sensory neurons (βIII-tubulin^+^) were significantly smaller, based upon distribution of diameters, than wild-type counterparts (*n*=4992 neurons, wild type, 4 ganglia/4 pups/4 litters; 5675 neurons, *LgDel*, 5 ganglia/5 pups/4 litters; *P*<0.0001, two-way ANOVA). (D) *LgDel* TrkB^+^ CNgV sensory neurons were significantly smaller than wild-type counterparts (*n*=100 TrkB^+^ neurons, wild type, 4 ganglia/2 pups/litters; *LgDel*, 4 ganglia/2 pups/litters; *P*≤0.009, two-way ANOVA). (E) *LgDel* TrkA^+^ CNgV sensory neurons were significantly smaller than wild-type counterparts (*n*=100 TrkA^+^ neurons, wild type, 4 ganglia/4 pups/2 litters; *LgDel*, 4 ganglia/4 pups/3 litters; *P*≤0.004, two-way ANOVA). (F) Expression levels of genes associated with mechanosensory (*MafA*) or nociceptive (*Ret*, *TrpV1*, *Scn10a* and *Scn11a*) neurons increased in *LgDel* P8 CNgV based upon qPCR analysis of five ganglia per genotype. The red bar for the MafA data and green bars for the Ret, TrpV1m, Scn10a and Scn11a data indicate the mean expression values for each transcript in each genotype. (G) Ret^+^ neurons were a subset of TrkA^+^ sensory neurons (G1-G4) in wild-type P8 CNgV. Fascicles of Ret^+^ axons (arrowheads, G,G1) were adjacent to TrkA^+^ axon fascicles that course among nodules of CNgV sensory neurons. (H) *LgDel* Ret^+^ CNgV sensory neurons had a similar distribution and coincidence with TrkA^+^ sensory neurons (H1-H3). (I) In register with increased *Ret* expression levels, Ret^+^ neurons in *LgDel* were significantly larger than wild-type counterparts (*n*=250 Ret^+^ cells, wild type and *LgDel*; 5 ganglia/5 pups/4 litters, wild type; 5 ganglia/5 pups/4 litters; **P≤0.0001, two-way ANOVA). (J,K) Similar proportions of TrkA^+^ cells were *Wnt1^Cre^*^+^ in wild-type and *LgDel* P8 CNgV. *Wnt1^Cre^*/^+^ TrkA^+^ neurons accounted for a substantial subset of the TrkA^+^ population in both genotypes (J1-J3,K1-K3). Quantification of these frequencies indicated that they were indistinguishable in the two genotypes (*n*=892 wild-type cells, 1007 *LgDel* cells, 2 ganglia, 1 pup per genotype). (L,M) TrpV1^+^/*Wnt1^Cre^*^+^ cells constituted a modest proportion of TrpV^+^ cells in wild-type (L) and *LgDel* (M) CNgV. The coincidence of Trpv1 and *Wnt1^Cre^* labeling was rare, and tended to occur in cells less intensely labeled for TrpV1 (asterisks, L1-L3,M1-M3). Approximately 23% of wild-type P8 CNgV TrpV1^+^ and 34% of *LgDel* TrpV1^+^ neurons were also *Wnt1^Cre^*^+^ (*n*=294 cells, 1 ganglion, 1 pup, wild type; *n*=189 cells, 1 ganglion, 1 pup, *LgDel*).

Altered progenitor division and neurogenesis in *LgDel* during early development may influence the outcome of CNgV differentiation. Thus, we evaluated divergence in CNgV sensory neuron classes at P8, when oropharyngeal dysfunction, including S/F/S disruption, is detected in *LgDel* pups (Karpinski et al., 2014; Yitsege et al., 2020). In wild-type CNgV, TrkB^+^-presumed mechanosensory cells were a minority of βIII-tubulin-labeled neurons (20%; Fig. 7A,D), whereas TrkA^+^ neurons constituted the majority (70%; Fig. 7B,E; see also Fig. 2), with an additional 10% of βtubulin-III neurons not labeled by either marker (*n*=4 wild-type ganglia, 4 P8 pups, 2 litters). These proportions did not differ in *LgDel*: 23% of CNgV sensory neurons expressed TrkB, whereas 65% expressed TrkA, and 12% were not labeled by either marker (*n*=4 ganglia, 4 *LgDel* P8 pups, 2 litters; *P*=0.50 TrkB^+^; *P*=0.64, TrkA^+^, *t*-test). There was no significant difference in the expression levels of *TrkB* or *TrkA* mRNA measured by qPCR in wild type versus *LgDel* CNgV (data not shown), and expression levels of the glial marker GFAP, which identifies satellite glia, measured by qPCR, were also equivalent in the two genotypes. Sensory neuron size is another fundamental metric of mechanoreceptive versus nociceptive classes; mechanoreceptive neurons on average are larger than nociceptive neurons (Le Pichon and Chesler, 2014). Mean areas and equivalent diameters of *LgDel* βIII-tubulin^+^ CNgV sensory neurons were significantly smaller than wild-type counterparts (Fig. 7C), reflecting a reduction of cell sizes in TrkB^+^ (Fig. 7D) and TrkA^+^ (Fig. 7E) subpopulations.

We next investigated whether diminished sizes of CNgV mechanosensory and nociceptive neurons are paralleled by altered expression levels of additional markers associated with subsets of mature mechanosensory neurons: *MafA* (Bourane et al., 2009); or nociceptive neurons *Ret*, *TrpV1*, *Scn10a (Nav1.8)*, *Scn11a (Nav1.9)* (Bennett et al., 2019; Lopes et al., 2012). The expression levels of all of these sensory neuron-associated genes, measured by qPCR, increased in *LgDel* P8 CNgV (Fig. 7F), suggesting that although cell proportions were apparently unchanged, transcriptional states were altered. To assess cellular correlates of this enhanced expression, we labeled P8 wild-type and *LgDel* CNgV for Ret (Fig. 7G,H), which recognizes a subset of TrkA^+^ sensory neurons. *LgDel* Ret^+^ sensory neurons were significantly larger than wild-type counterparts (Fig. 7I; *n*=5 ganglia/5 embryos/5 litters, 250 cells total, wild type; 5 ganglia/5 embryos/5 litters, 250 cells total, *LgDel*), perhaps in register with increased levels of *Ret* mRNA.

To further define cellular diversity in P8 *LgDel* and wild-type CNgV, we compared relationships between *Wnt1^Cre^*^+^ sensory neurons at P8 with markers of nociceptor identity: TrkA (Fig. 7J,K) and TrpV1 (Fig. 7L,M). The proportions of TrkA^+^ versus all sensory neurons were similar in P8 *LgDel* and wild-type CNgV, as were the levels of *TrkA* transcripts (see above). If TrkA^+^/*Wnt1^Cre^*^+^ cells within the TrkA^+^ subpopulation differentiate in parallel in the two genotypes, one would expect no distinction in the frequency of *Wnt1^Cre^*^+^/TrkA^+^ cells. An apparently stable proportion of CNgV TrkA^+^ cells [38% (wild type) and 36% (*LgDel*)] were *Wnt1^Cre^*^+^ at P8 (*n*=892 cells, wild type; *n*=1002 cells, *LgDel*; two sections each from one P8 pup of each genotype). In contrast, 23% of TrpV1^+^ sensory neurons in wild type were *Wnt1^Cre^*^+^ versus 34% in *LgDel* (*n*=1276 cells, wild type; *n*=1114, *LgDel*; two sections each from one P8 pup of each genotype)*.* Although not statistically definitive, this potential distinction is consistent with a potential parallel change of *Wnt1^Cre^*^+^/Trpv1^+^ cell proportions and *TrpV1* transcript levels in *LgDel* versus WT P8 CNgV.

## DISCUSSION

**Fig. 8.**
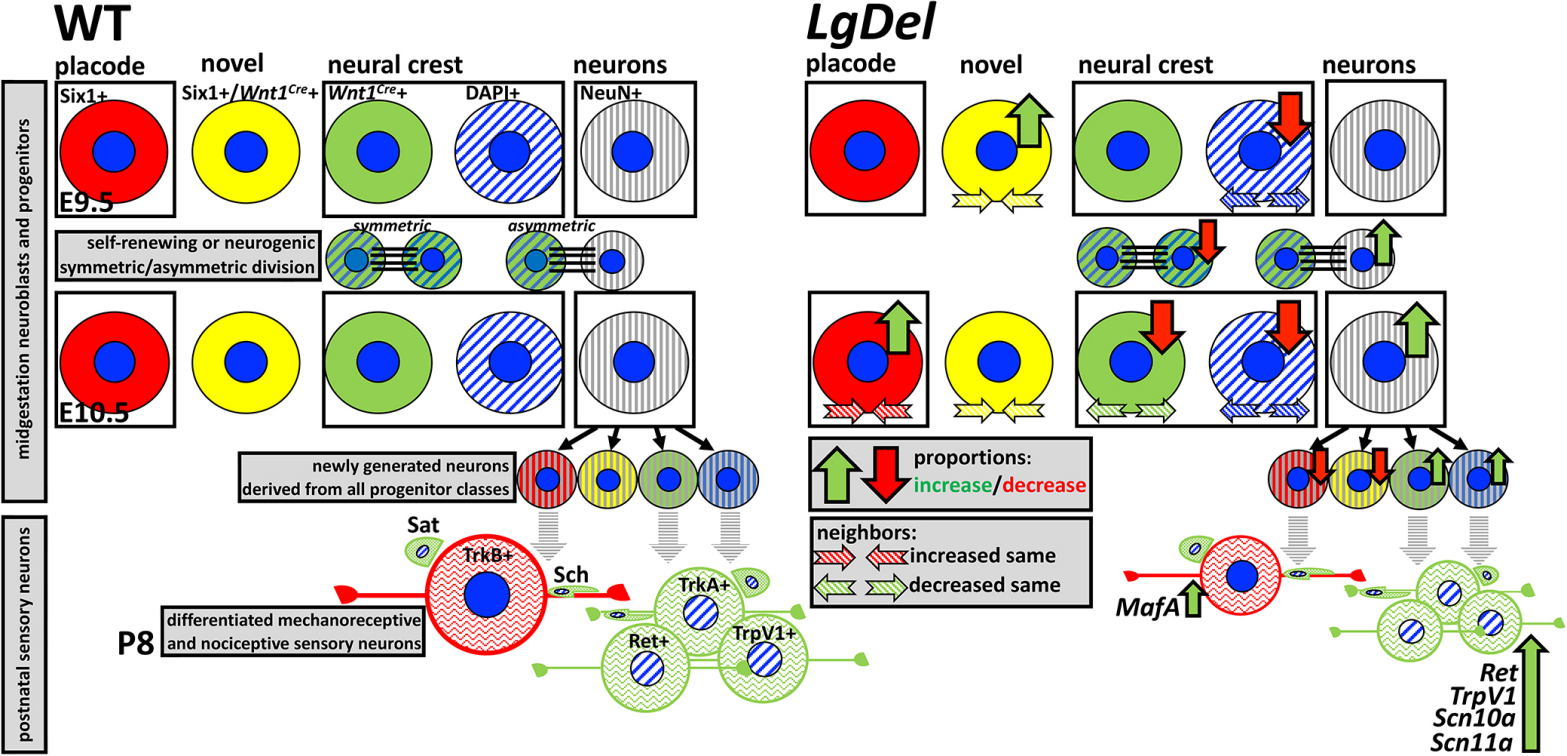
**Divergent progenitor regulation and sensory neuron differentiation distinguish *LgDel* CNgV.** The left panel shows that in wild type (WT), at E9.5, there are four distinct trigeminal progenitor classes: Six1^+^ placode-derived (red); a novel class that are labeled by Six1 and the *Wnt1^Cre^* lineage reporter (yellow); *Wnt1^Cre^*^+^ neural crest-derived (green), DAPI^+^ (Wnt1^−^) neural crest-derived (blue hatched) and postmitotic neuroblasts (NeuN^+^; gray hatched). Each class is seen at E10.5, at which time we identified symmetric and asymmetric self-renewing, as well as neurogenic modes of division among presumed neural crest progenitors. At P8, the *Wnt1^Cre^*^+^ subset gives rise primarily to TrkA^+^, Ret^+^ and Trpv1^+^ presumed nociceptive neurons, and subsets of satellite and Schwann cells. We infer that the primary source of the TrkB^+^ presumed mechanoreceptors is the Six1^+^ precursor population; however, current data do not definitively demonstrate this inference. The right panel shows that in *LgDel*, by E10.5, proportions of placode-derived cells increase and neural crest-derived cells decrease, in register with increased asymmetric neurogenic rather than self-renewing progenitor divisions of neural crest-derived cells. In parallel, neighbor relationships of each progenitor class change from equivalent in wild-type to Six1^+^ progenitors being more likely, and neural crest progenitors less likely, to have neighbors of the same class. These changes are accompanied by increased newly generated NeuN^+^ neurons distributed across Six1^+^, Six1^+^/*Wnt1^Cre^*^+^, *Wnt1^Cre^*^+^ or DAPI^+^ classes. At P8, proportions of TrkA^+^ and TrkB^+^ sensory neurons are similar in *LgDel* and wild type; however, sizes are significantly smaller in *LgDel*. In addition, expression levels of several markers, especially those associated with nociceptors (far right), are increased. Thus, divergent proportions, neighbor relations, modes of division and neurogenesis from distinct progenitor populations prefigure divergent CNgV sensory neuron differentiation.

Proportions of neural crest- versus placode-associated progenitors, cell-cell interactions and modes, as well as the frequency of neurogenic divisions, diverge during early CNgV development in the *LgDel* mouse 22q11.2 DS model. These changes result in heterochronic neurogenesis from primarily neural crest progenitors, and prefigure altered early postnatal CNgV sensory neuron differentiation (Fig. 8). The deletion of 22q11 apparently results in altered sizes and levels of gene expression for molecularly defined classes of CNgV sensory neurons, with many of these changes focused on presumed neural crest-derived nociceptive neurons (Fig. 8). Thus, neural crest-derived CNgV progenitors are a target for quantitative disruption of peripheral orofacial sensory development due to heterozygous 22q11.2 deletion. This early quantitative disruption of progenitor characteristics prefigures divergent sensory neuron identity that could influence sensory motor coordination for a number of orofacial behaviors, including S/F/S, disrupted in 22q11DS.

### Analyzing precursor diversity, neuronal diversity and quantitative change

We used quantitative genetic/molecular cytological analyses to define basic mechanisms of CNgV progenitor proliferation and division, as well as sensory neuron differentiation and diversity in wild-type and *LgDel* mice carrying a heterozygous deletion that accurately models 22q11.2DS, a common human genetic/developmental disorder (McDonald-McGinn et al., 2015; Meechan et al., 2015a; Motahari et al., 2019). The differences we identified were not readily apparent based upon qualitative microscopic inspection; they were only recognized by quantifying proportions, positions and neighbor relations of cells with multidimensional molecular identities in different proliferative states in multiple samples. Variation between wild type versus *LgDel* individuals requires adequate sampling and quantification to identify distinctions (Maynard et al., 2008; Meechan et al., 2007, 2015a, 2006, 2009; Motahari et al., 2019). It is likely that additional phenotypes in a broad range of neurodevelopmental disorders, especially those associated with copy number variants or polymorphic alleles, reflect similarly subtle variable changes of early development that have, as yet, escaped detection. Our data suggest that such early developmental divergence can influence neuronal differentiation, neural circuit organization and behavior.

### Placode versus Crest contributions to 22q11 gene-dependent CNgV development

The initial placodal derivation of CNgV progenitors for mechanoreceptive sensory neurons (Ayer-Le Lievre and Le Douarin, 1982; D'Amico-Martel and Noden, 1983; Hamburger, 1961), and migration of neural crest-derived progenitors that generate nociceptive neurons (George et al., 2010; Karpinski et al., 2016; Liebl et al., 1997; Martin-Zanca et al., 1990), are not noticeably disrupted, and initial CNgV coalescence does not differ substantially in *LgDel* versus wild-type embryos. Instead, 22q11 deletion alters neighbor relationships of placode- and neural crest-derived progenitors once CNgV forms. We confirmed previous work, undertaken in several species (Aguillon et al., 2016; Baker and Bronner-Fraser, 2000; Blentic et al., 2011; Karpinski et al., 2016), that most placode-derived precursors undergo terminal neurogenic division by midgestation, before terminal divisions of neural crest-derived progenitors. We identified additional molecular diversity within these populations. The proportionate expansion and increased proliferative frequency of E9.5 *LgDel* CNgV Six1^+^/*Wnt1^Cre^*^+^ cells identifies these molecularly defined progenitors as a potential target for selective disruption. Quantitative changes in proportions of Six1^+^/*Wnt1^Cre^*^+^ cells do not conform fully to those in the Six1^+^, *Wnt1^Cre^*^+^ or DAPI^+^ classes. Thus, this population has a unique developmental profile that is neither that of presumed placode- (Six1^+^) or neural crest-derived (*Wnt1^Cre^*^+^ or DAPI^+^) cells. We cannot definitively resolve placode versus neural crest derivation of Six1^+^/*Wnt1^Cre^*^+^ cells at this time. Their distinct behavior in wild type versus *LgDel* CNgV, and identification of similar cells in the other cranial sensory ganglia (Karpinski et al., 2016), indicates they are likely a distinct class, and that there is additional diversity among progenitors beyond placode versus neural crest lineage distinctions.

A significant proportion of neural crest-derived *Wnt1^Cre^*^+^ and DAPI^+^ cells are proliferative in E10.5 *LgDel* and wild-type CNgV, after most Six^+^ placode-associated cells are postmitotic. Previous work (Steventon et al., 2014) indicates that early signaling between placode and neural crest cells regulates subsequent proliferative and neurogenic capacities, especially for initial cranial neural crest populations. The deletion of 22q11 may alter similar interactions between later generated progenitors of diverse lineages during gangliogenesis. Our transcriptome analyses suggest that 22q11 deletion modifies expression of cell surface molecules, signals, adhesion molecules and effectors that influence CNgV differentiation (Jourdeuil and Taneyhill, 2020; Lassiter et al., 2014; Shiau and Bronner-Fraser, 2009; Shigetani et al., 2008; Voelkel et al., 2014; Wakamatsu et al., 2000). Together, these data indicate that divergent cell-cell interactions between neural crest-derived precursors of distinct sublineages and trigeminal placode-derived neighbors in *LgDel* may underlie anomalous modes of cell division and premature CNgV neurogenesis.

### Altered neurogenesis and its consequences in CNgV at midgestation

Proportions of CNgV placode- and neural crest-derived progenitors or neurons diverge for *LgDel* versus wild type by E9.5 and continue to change at E10.5, primarily via accumulation of postmitotic neurons in *LgDel* generated by neural crest precursors. Thus, *LgDel* CNgV cranial neural crest cells seemed a likely target for 22q11 deletion-mediated pathology. We found increased asymmetric neurogenic divisions of E10.5 *LgDel* neural crest precursors. This change in neurogenic capacity of intermediate CNgV progenitors may parallel the disruption of *LgDel* cerebral cortical basal progenitors (Meechan et al., 2015b, 2009). The consequences of intermediate progenitor disruption for CNgV differentiation may be substantial and sustained. The capacity of subsets of sensory neuron progenitors to respond to local signals, including neurotrophic factors (elshamy and Ernfors, 1996; Snider, 1994), could be altered, potentially eliminating precursor subsets with particular fate biases, similar to aberrant differentiation of TrkC^+^ (also known as Ntrk3) sensory neuron precursors in NT-3 knockout mice (Farinas et al., 1996). In *LgDel* CNgV, similar disruption could alter the balance of mechanoreceptive versus nociceptive peripheral and central projections. This could lead to the discoordination of local peri-oral motor control due to ambiguous sensory representations and sensory/motor integration.

Premature genesis of sensory neurons, due to aberrant history or mode of division (Smith et al., 2017; Sunchu and Cabernard, 2020), could alter interactions between differentiating neurons. Anomalously early generated *LgDel* CNgV neurons could extend axons aberrantly to inappropriate peripheral or central targets (Motahari et al., 2020). There may be an optimal wild-type distribution of CNgV mechanosensory and nociceptive neurons, similar to ‘tiling’ (Grueber and Sagasti, 2010; Lefebvre et al., 2015; Mountoufaris et al., 2018) of neuron classes that influences distribution of axons in other sensory systems. Thus, divergent neighbor relations of placode- versus neural crest-associated progenitors or neurons in *LgDel* CNgV could substantially disrupt subsequent peripheral and central innervation. Such changes can alter innervation patterns and function in other sensory systems (Petrovic and Schumcker, 2015). The 22q11 deletion-dependent disordered trigeminal sensory innervation of perioral and pharyngeal structures may contribute to S/F/S difficulties due to 22q11 deletion. These disruptions of trigeminal sensory circuits could also lead to continued deficits, including dysarthria or other sensory/motor speech disruptions in children and adults with 22q11DS (Baylis and Shriberg, 2019; Persson et al., 2017).

### Changes in mature *LgDel* CNgV sensory neurons

The multilobular geometry of P8 CNgV – sensory neuron and satellite glial aggregates intercalated with axon fascicles – complicates the quantitative assessment of sensory neuron subclasses (Bennett et al., 2019; Le Pichon and Chesler, 2014). Thus, direct comparisons of early quantitative changes and those in mature CNgV are not possible. We identified some aspects of sensory neuron differentiation that may reflect quantitative divergence of cell-cell interactions and neurogenesis in midgestation *LgDel* CNgV. *LgDel* P8 CNgV sensory neuron anomalies are predominantly associated with neural crest-derived nociceptive cells (Fig. 8). These changes could modify nociceptive oropharyngeal sensation in *LgDel*, disrupting accuracy or utility of sensory information that optimizes orofacial behaviors. Distorted detection of aversive stimuli during milk intake, or in response to aspiration, may alter S/F/S acutely. Alternatively, sensitivity to aversive stimuli may be increased by chronic inflammation caused by aspiration and infection associated with suboptimal S/F/S (LaMantia et al., 2016; Maynard et al., 2020b). Thus, the neural crest/nociceptor-focused changes in *LgDel* P8 CNgV could alter reflex control, relay of distinct dermal stimuli and motor control of CN V-dependent oromotor behaviors (Karpinski et al., 2014; Welby et al., 2020).

### Early developmental disruption and pathological consequences

Mouse models of neurodevelopmental disorders have mostly been used to define ‘end stage’ pathology that substantially compromises behavior, circuit organization/function and synaptic activity in mature animals (Nakai et al., 2018; Sohal and Rubenstein, 2019; Telias, 2019; Verma et al., 2019). Less attention has been paid to disrupted developmental trajectories that may lead to mature pathology, in part because relevant phenotypes are difficult to detect in embryos in which cell biological and morphogenetic changes are rapid and transient. Our analysis of CNgV development in the *LgDel* 22q11DS mouse model demonstrates how defining these quantitative changes during early embryonic development can contribute to the identification of subsequent neural circuit and behavioral pathology. Our data indicate that early changes in progenitor proportions, neighbor relations and modes of cell division largely focused on neural crest-derived cells during CNgV genesis. This early divergence prefigures changes in the mature CNgV, focused particularly on presumed nociceptive cell classes derived from the neural crest. These early errors in CNgV cellular architecture and neurogenesis due to 22q11 deletion may reflect a larger systemic disruption of an optimal developmental program that begins with patterning the rhombencephalon and early craniofacial and cranial nerve morphogenesis. These initial changes, concatenated with subsequent developmental divergence, ultimately lead to altered circuits that fail to support optimal S/F/S in *LgDel* pups (Karpinski et al., 2014; Yitsege et al., 2020). Defining the earliest developmental antecedents of circuit disruption, even if subtle and statistical, may be essential for understanding neurodevelopmental pathology and establishing optimally effective interventions to ameliorate specific S/F/S deficits.

## MATERIALS AND METHODS

### Animals

The George Washington University Animal Research Facility maintained colonies of wild-type C57/BL6N (Charles River Laboratories), *LgDel* mice (Merscher et al., 2001), *Wnt1^Cre^* (Chai et al., 2000), Rosa26-GFP-NLS-lacZ cre-reporter mice (GNZ; Stoller et al., 2008) and *Sox10-H2B^Venus^* transgenic reporter mice (Corpening et al., 2011) on the same isogenic C57/BL6N background. We used the C57/BL6N strain to avoid complications due to a mutation in the C57/BL6J strain that may enhance effects of 22q11 deletion (Ronchi et al., 2013). The *LgDel* mutation, *Wnt1^Cre^* driver and *Sox10^Venus^* reporter were transmitted paternally; Rosa26-GFP-NLS-LacZ was transmitted maternally. For proliferation analyses, BrdU (50 mg/kg body weight) was injected into timed pregnant mice at E10.5 and embryos were harvested 2 h after labeling. Timed pregnant females (vaginal plug=E0.5) were sacrificed at E9.5 or E10.5 by rapid cervical dislocation and embryos collected for analysis. The George Washington University Institutional Animal Care and Use Committee approved all procedures.

### Immunohistochemistry and microscopy

E9.5 and E10.5 embryos were immersion fixed and prepared for cryosectioning and antibody labeling as described previously (Karpinski et al., 2016). At P8, each CNgV was dissected after aldehyde perfusion fixation, and then prepared and embedded whole for cryosectioning. The primary antibodies used were mouse anti-βIII tubulin (BioLegend, 801201, 1:1000), rabbit anti-Six1 (Proteintech, 10709, 1:1500), rabbit anti-fibronectin (Millipore, AB2033, 1:1000), anti-cleaved caspase 3 (Cell Signaling Technology, 9661, 1:200), chicken anti-GFP (Abcam, ab13970, 1:1000), mouse anti-NeuN (Merck Millipore, MAB377, 1:1000), rabbit anti-NeuN (Cell Signaling Technology, 24307, 1:400), mouse anti-BrdU (BD Biosciences, 555627, 1:100), rat anti-BrdU (Novus, NB500-169, 1:100), rabbit anti-Sox2 (Stemgent, 09-0024, 1:100), goat anti-TrkB (R&D Systems, AF1494, 1:100), anti-TrkA (Alomone Labs, ANT-018, 1:100), goat anti-Ret (Neuromics, GT15002, 1:50) and rabbit anti-TrpV1 (Alomone Labs, ACC-030, 1:100). Primary antibody labeling was visualized using Alexa Fluor 488-, 54- or 647-conjugated secondary antibodies (Molecular Probes, 1:4000, 488; 1:2000, 546 and 1:1000, 647). Standard BrdU immunolabeling techniques were used after acid treatment for antigen retrieval. Images were collected on a Leica Tiling or a Zeiss 710 confocal microscope.

### Imaging and analysis

Cells were counted in images of sections from each ganglion collected at 20× magnification on a Leica Tiling microscope (embryonic CNgV) or a Leica TCS SP8 multiphoton scanning confocal microscope (P8 CNgV). Red (Six1), green (*Wnt1^Cre^*/*Rosa26-GNZ*; abbreviated as *Wnt1^Cre^* throughout the text), blue (DAPI) and infrared (Alexa-Fluor 647) channels were visualized separately and superimposed as composite images (*Wnt1^Cre^*::*Rosa26-GNZ* signal was amplified for imaging using anti-eGFP antibody labeling). Labeled cells were counted as previously described (Karpinski et al., 2016). To determine proportions of proliferating cells or differentiating neurons in embryonic material, numbers of cells positive for BrdU or NeuN were assessed for Six1, *Wnt1^Cre^*(GFP), Six1/ *Wnt1^Cre^* or DAPI labeling, and percentages were calculated. To assess TrkB, TrkA, Ret or TrpV1-labeled neuron subpopulations, fields were counted for all βIII-tubulin^+^ neurons, followed by those co-labeled for TrkB/TrkA/Ret/Trpv1, and percentages were determined. Chi-Square/Fisher exact tests, *t*-tests or ANOVA were used to assess statistical differences between genotypes and cell classes as described for each experiment in the Results.

### Neighbor analysis

To calculate the nearest neighbor statistics, images of immunofluorescently labeled cryosections were captured, and cells of each class were marked in separate layers in Adobe Photoshop. These layers were imported into ImageJ, and XY coordinates for each labeled cell were computed using the analyze particle function. Using Microsoft Excel, the nearest neighbor for each cell was calculated by computing the distance between every cell in the ganglia and identifying the closest neighbor by identifying the pairing with the minimum value of the computed distance. Frequency percentages were then calculated for each ganglia, and statistical significance was computed by two-way ANOVA and individual comparisons were computed by Holm–Sidak's post-hoc analysis.

### Pair cell assay

CNgV were microdissected from entire E10.5 litters (5 independent experiments/5 litters, wild type; *n*=4 independent experiments/4 litters, *LgDel*) and dissociated as described previously (Lehtinen et al., 2011; Shen et al., 2002; Tucker et al., 2010). Dissociated cells were plated at clonal density (35 cells/µl; 14 µl total volume/well) on poly-D-lysine-coated Terasaki plates. Cultures were incubated for 21 h at 37°C with 5% CO_2_, fixed and immunolabeled for Sox2 (progenitor marker) and βIII-tubulin (neuronal marker), as well as DAPI to identify nuclei. Pairs of cells were identified based upon DAPI labeling in individual wells based upon apposition of two cells isolated from any other cells. For each isolated DAPI-labeled pair, the expression of progenitor and neural markers was visualized and scored.

### Ganglion volume and cell density calculation

Ganglion volume measurements were made on E10.5 embryos as described previously (Pomeroy et al., 1990). Serial sections (16 µm) were prepared from the entirety of each ganglion and immunostained with antibodies to fibronectin and βIII tubulin. The area of each CNgV section was measured by tracing its outline in Image J and converting pixels to µm^2^. These areas were then multiplied by section thickness for volume, and all volumes added together. For cell density measurements, ganglia were outlined to determine areas, pixel numbers were converted to microns, and numbers of DAPI-labeled cells per ganglia were counted and the number of cells was divided by ganglion area.

## Supplementary Material

Supplementary information
