# Supplementary material for: Selective disruption of trigeminal sensory neurogenesis and differentiation in a mouse model of 22q11.2 deletion syndrome
Source: Dis Model Mech. 2021 May 4;15(2):dmm047357. doi: 10.1242/dmm.047357 (PMC8126478; doi:10.1242/dmm.047357)
Supplement: Supplementary information [file dmm-15-047357-s1.pdf]

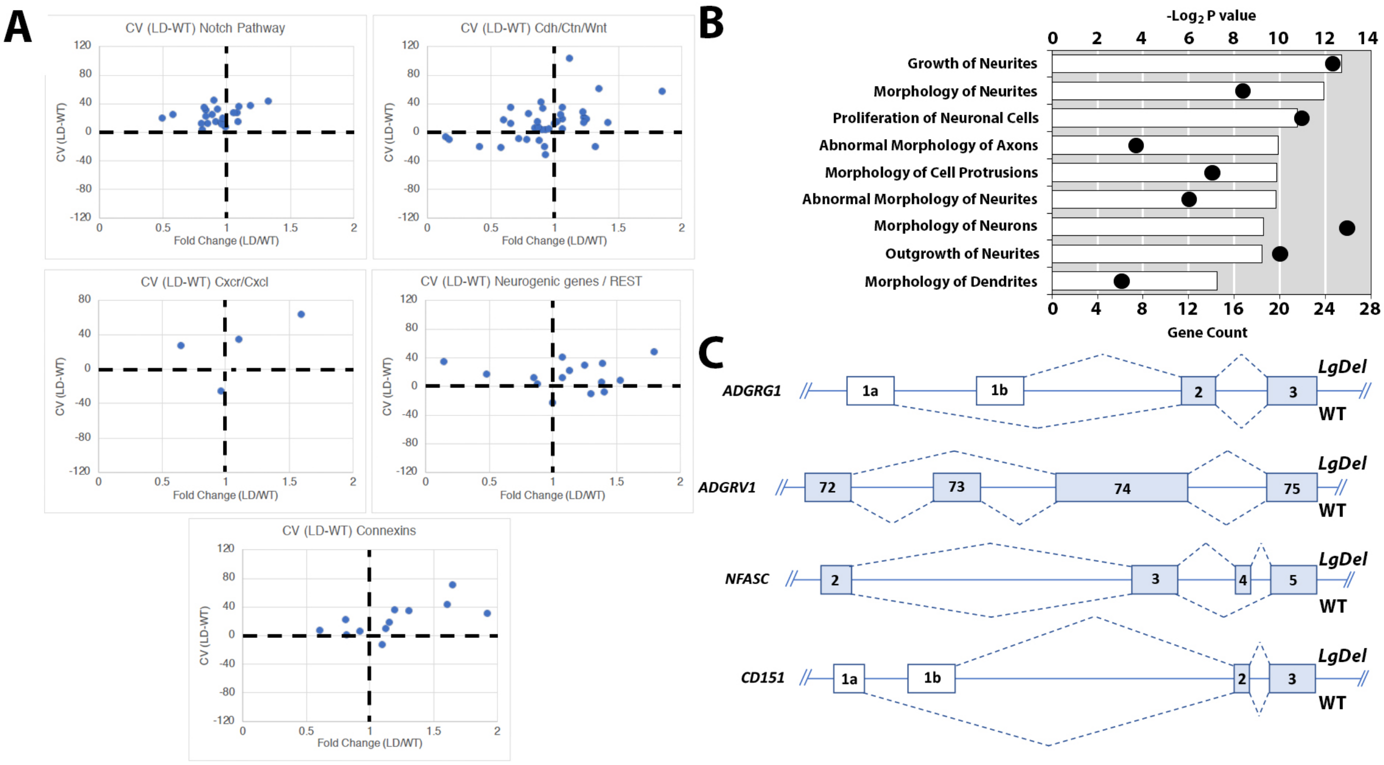

Figure S1: Differential expression, variation and splicing of genes mediating cell-cell interaction and adhesion in *LgDel* CNgV. **A**) Differential expression levels plotted as a function of coefficient of variation for *Notch*, *Cxcl/Cxcr*, *Connexin/Gja*, and *Wnt/Cadherin/Catenin* gene families. Dotted horizontal lines indicate the threshold for coeffcicient of varation *LgDel* > WT (above) versus WT> *LgDel* (below), and dotted vertical lines indicate differential expression of transcripts (left, *LgDel*<WT; right *LgDel*>WT). **B**) Biological processes significantly enriched with genes undergoing differential mRNA splicing in a comparison of *LgDel* versus WT. The bars indicate biological process and P value. The circles indicate numbers of genes undergoing differential mRNA splicing in the biological process. **C**) Alternative RNA splicing in *LgDel* of genes encoding transmembrane proteins involved in cell Adhesion and neurite morphology. Schematic representation of alternative RNA splicing events observed in 4 genes. Open boxes represent 5’ untranslated regions (UTR). Closed boxes represent open reading frames. NMD, nonsense mediated decay. *Adgr1* and *Cd151/tetraspanin* splice variants result in alternative 5’UTRs. *Adgrv1* alternative splicing results in exon skipping leading to nonsense-mediated decay. Nfasc alternative splicing results in in-frame of Exon 4.
